# Decoding Cortical Glial Cell Development

**DOI:** 10.1007/s12264-021-00640-9

**Published:** 2021-02-19

**Authors:** Xiaosu Li, Guoping Liu, Lin Yang, Zhenmeiyu Li, Zhuangzhi Zhang, Zhejun Xu, Yuqun Cai, Heng Du, Zihao Su, Ziwu Wang, Yangyang Duan, Haotian Chen, Zicong Shang, Yan You, Qi Zhang, Miao He, Bin Chen, Zhengang Yang

**Affiliations:** 1 State Key Laboratory of Medical Neurobiology, Institutes of Brain Science, MOE Frontiers Center for Brain Science, Department of Neurology, Institute for Translational Brain Research, Zhongshan Hospital, Fudan University, Shanghai, 200032 China; 2 Department of Molecular, Cell and Developmental Biology, University of California Santa Cruz, Santa Cruz, CA 95064 USA

**Keywords:** Radial glial cell, Intermediate progenitor cell, ASCL1, EGFR, OLIG2, Oligodendrocyte, Astrocyte, Olfactory bulb interneuron, Cerebral cortex

## Abstract

Mouse cortical radial glial cells (RGCs) are primary neural stem cells that give rise to cortical oligodendrocytes, astrocytes, and olfactory bulb (OB) GABAergic interneurons in late embryogenesis. There are fundamental gaps in understanding how these diverse cell subtypes are generated. Here, by combining single-cell RNA-Seq with intersectional lineage analyses, we show that beginning at around E16.5, neocortical RGCs start to generate ASCL1^+^EGFR^+^ apical multipotent intermediate progenitors (MIPCs), which then differentiate into basal MIPCs that express ASCL1, EGFR, OLIG2, and MKI67. These basal MIPCs undergo several rounds of divisions to generate most of the cortical oligodendrocytes and astrocytes and a subpopulation of OB interneurons. Finally, single-cell ATAC-Seq supported our model for the genetic logic underlying the specification and differentiation of cortical glial cells and OB interneurons. Taken together, this work reveals the process of cortical radial glial cell lineage progression and the developmental origins of cortical astrocytes and oligodendrocytes.

**Supplementary Information:**

The online version of this article (10.1007/s12264-021-00640-9) contains supplementary material, which is available to authorized users.

## Introduction

The mammalian cerebral cortex, controlling the highest brain functions, contains billions of neurons and glia. During mouse cortical development, radial glial cells (RGCs), known as primary neural stem cells (NSCs), give rise to distinct subtypes of cortical neurons, known as glutaminergic projection pyramidal neurons (PyNs) and cortical oligodendrocytes, astrocytes, and olfactory bulb (OB) gamma-aminobutyric acid (GABAergic) interneurons [1]. Although some PyNs are directly derived from RGCs and some RGCs can directly transform into astrocytes [2–4] and ependymal cells [5], many lines of evidence suggest that most neurons, oligodendrocytes, and astrocytes are not the direct progeny of RGCs, but instead originate from intermediate progenitor cells (IPCs) [1], highlighting the important role of IPCs. During the period of cortical neurogenesis around E11.5-E16.5, RGCs undergo asymmetric cell divisions to self-renew and to produce PyN-IPCs, which exclusively generate PyNs in an inside-out pattern: deep layer PyNs are born first, followed by PyNs of upper layers [1, 6–9]. Transcription factors that control the generation of diverse types of PyNs in different cortical layers with unique properties have been identified [10, 11].

At the late embryonic stage, cortical RGCs undergo a major switch in their progenitor properties and produce cortical oligodendrocytes, astrocytes, and OB interneurons (OBiNs) [1, 12, 13]. Oligodendrocytes and astrocytes are macroglial cells, the most abundant cell type in the cortex. We know that these glial cells perform key functions vital to the physiology of the cerebral cortex. However, we do not know how cortical oligodendrocytes, astrocytes, and OBiNs are produced from RGCs; especially, a precise understanding of astrocyte lineage-restricted progenitor cells (AS-IPCs) and its developmental process are lacking.

Here, by combining single-cell RNA-Seq with intersectional lineage analyses, we found that mouse cortical RGC-derived oligodendrocyte cell lineage (OL-lineage), astrocyte cell lineage (AS-lineage), and OBiN cell lineage (OBiN-lineage) can be identified in the neonatal brain; they are mainly derived from multipotent IPCs (MIPCs). Briefly, around E16.5, cortical RGCs start to generate ASCL1^+^EGFR^+^ apical MIPCs (aMIPCs) in the ventricular zone (VZ) and subventricular zone (SVZ). aMIPCs quickly differentiate into basal MIPCs (bMIPCs) that express ASCL1, EGFR, OLIG2, and MKI67. bMIPCs undergo several rounds of divisions to generate cortical oligodendrocytes, astrocytes, and OBiNs. Interestingly, those RGCs that are translocating to the cortex and transforming into AS-IPCs in late embryogenesis also express ASCL1, EGFR, and OLIG2. Finally, single-cell ATAC-Seq (scATAC-Seq) supported our model for the genetic logic underlying the specification and differentiation of cortical glial cells and OB interneurons. Taken together, this work reveals the process of cortical RGC lineage progression and the developmental origins of cortical astrocytes and oligodendrocytes.

## Materials and Methods

### Mice

All experiments performed at Fudan University Shanghai Medical College were approved by Fudan University Animal Ethics Committee. We generated the *Ascl1*^*Flpo*^ allele by inserting a *P2A-Flpo-P2A-tTA* DNA cassette immediately before the stop codon of the *Ascl1* gene (Fig. 6A), using a CRISPR/Cas9-based strategy. The sgRNAs, the targeting vector, and Cas9 were injected into C57BL/6 zygotes to generate founder mice, which were screened by PCR to detect integration of the targeting vector. Genomic DNA from mice with positive integration was used to amplify the integration junction for confirmation by sequencing. Finally, southern hybridizations using both an *Flpo* probe and a 3’-probe were performed to confirm the correct targeting.

The generation and genotyping of the *HG-loxp* [14], *Olig2-tva-Cre *[15], *hGFAP-GFP* [16], *Ai65F* [14], and *IS* reporter [14] mice were as described previously. The day of vaginal plug detection was designated E0.5. The day of birth was designated P0. The genders of the embryonic and early postnatal mice were not determined. Both male and female mice were used.

### Immunohistochemistry

Immunohistochemistry was performed using standard protocols [13]. Brains were cryosectioned at 12 μm or 30 μm. The sections were first permeabilized with 0.05% Triton X-100 for 30 min, followed by incubation in blocking buffer (5% donkey serum and 0.05% Triton X-100 in TBS) for 2 h. The blocking buffer was removed, and the sections were incubated with primary antibodies (diluted in blocking buffer) overnight at 4°C. The sections were washed in TBS and incubated with secondary antibodies conjugated to Alexa Fluor488, Cy2, Cy3, or Cy5 for 1 h at room temperature. The secondary antibodies were from Jackson ImmunoResearch and Invitrogen. Finally, the sections were counterstained with DAPI for 3 min before being mounted in fluorescence mounting medium (DAKO S3023).

We used the following primary antibodies: GFP (1:5,000, chicken, Aves Labs GFP-1020), tdTomato (1:2,000, goat, SICGEN Ab8181), ASCL1 (1:3,000, rabbit, Cosmo Bio SKT01-003), SP8 (1:5,000, goat, Santa Cruz Sc-104661), MKI67 (1:1,000, antigen retrieval, mouse, BD Pharmingen 556003), OLIG2 (1:1,000, rabbit, Millipore AB9610), OLIG2 (1:1,000, antigen retrieval, mouse, Millipore MABN50), PDGFRA (1:1,000, rat, BD Pharmingen 558774), ID3 (1:5,000, rabbit, Biocheck BCH-4/17-3), EGFR (1:2,000, goat, R&D System AB_355937), ALDH1L1 (1:1,000, rabbit, antigen retrieval, Abcam AB_10712968), SOX10 (1:500, goat, R&D System AB_442208), GFAP (1:1,500, rabbit, DAKO Z0334), and S100B (1:1,500, rabbit, DAKO Z0311).

### Image acquisition and analysis

Images for quantitative analyses were acquired using the Olympus FV1000 confocal microscope system, and cell counting was performed either on Z-stack confocal images or images from the Olympus VS120 Automated Slide Scanner. Statistical analyses were performed with GraphPad Prism 5.0, Microsoft Excel, and R language.

The percentages of translocating RGCs that expressed MKI67, ASCL1/EGFR/OLIG2, and ID3 in E17.5 brain sections from CD1 wild-type (*WT)* mice were calculated. We quantified 3–4 coronal sections from rostral to caudal levels of the telencephalic hemisphere per mouse, and 3 mice were analyzed. The percentages of EGFR^+^ cells that expressed MKI67 in the VZ, SVZ and intermediate (IZ) zones were calculated (*n* = 5 mice, 3 sections were counted per mouse). The percentages of OLIG2^+^ cells in the VZ and SVZ that expressed ASCL1 and EGFR were calculated (*n* = 4 mice, 3 sections were analyzed per mouse).

The percentages of GFP^+^ cells that were EGFR^+^, SOX10^+^, SP8^+^, ID3^+^, and ALDH1L1^+^ were calculated in somatosensory cortices of E18.5 *Ascl1*^*Flpo/Flpo*^; *IS* mice in which *pCAG-Cre* was electroporated into cortical VZ on E15.5. We counted the cortical cells in a 690-μm-wide field per section, in 3 sections per mouse (*n* = 4). The percentages of GFP^+^OLIG2^+^ cells (oligodendrocytes) and of GFP^+^S100B^+^ cells (astrocytes) in the P21 cortex (0.99 mm^2^) were calculated from 3-4 randomly-selected 30-μm sections per mouse (*n* = 3 mice). The percentages of GFP^+^ cells and tdT^+^ cells in the OB sections were calculated; we counted cells in a 0.12-mm^2^ area on each OB section; 2 sections per OB and 3 OBs were analyzed.

The percentages of GFP^+^OLIG2^+^ cells (oligodendrocytes) and of GFP^+^S100B^+^ cells (astrocytes) in the P21 cortex (1.2 mm^2^) were calculated from 3-4 randomly-selected 30-μm sections per *Olig2-tva-Cre; IS* mouse (*n* = 3). The percentages of H2B-GFP^+^ cells that were OLIG2^+^, SOX10^+^, S100B^+^, GFAP^+^, and ALDH1L1^+^ in the P21 cortices (0.6 mm^2^) were calculated from 3 randomly-selected 30-μm sections from P21 *Olig2-tva-Cre; HG loxp* mice (*n* = 3).

The percentages of OLIG2^+^ cells that expressed EGFR and ASCL1, those of EGFR^+^ASCL1^+^cells that expressed OLIG2, and those of EGFR^+^ cells that expressed MKI67 in the E16.5 CD1 *WT* cortical VZ and SVZ were calculated. Cells in the cortex were counted from 3 sections per mouse (*n* = 3). MKI67-expressing cells in the cortical VZ, SVZ, and IZ were counted (*n* = 5 mice, 3 sections per mouse). The percentages of OLIG2^+^ cells in the VZ and SVZ that expressed ASCL1 and EGFR were counted (*n* = 4 mice, 3 sections per mouse). The percentages of EGFR^+^ ID3^+^, ASCL1^+^, and EGFR^+^ MKI67^+^ cells in the cortical VZ, SVZ, IZ, and CP (cortical plate) were calculated in E18.5 WT cortex. Cells were counted from 3 randomly-selected 12-μm sections (*n* = 3).

The numbers of SOX10^+^, ALDH1L1^+^ and EGFR^+^ID3^+^ cells in the presumptive somatosensory cortices from *WT* mice aged E17.5 to P21 were counted. We counted cortical cells in a 350-μm width bin per section (*n* = 3 sections and 3 mice per group).

### *In utero* electroporation (IUE)

IUE of *Ascl1*^*Flpo/Flpo*^*; IS* or *Olig2-tva-Cre; IS* embryos was performed at E15.5. The plasmid *pCAG-Cre* (Addgene #13775) or *pCAG-Flpo* (Addgene #60662) (final concentration 1-2 μg/μL, 0.5 μL per embryo) was mixed with 0.05% Fast Green (Sigma), and injected into the lateral ventricle of embryos using a beveled glass micropipette. Five electrical pulses (duration: 50 ms) were applied at 38 V across the uterine wall with a 950-ms interval between pulses. Electroporation was performed using a pair of 7-mm platinum electrodes (BTX, Tweezertrode 45-0488, Harvard Apparatus) connected to an electroporator (BTX, ECM830). Embryos were analyzed at P21.

### scRNA-Seq library preparation

*pCAG-Cre* plasmids were electroporated into the cortical VZ of *IS* mice at E15.5. P1 pups were sacrificed, and the brains were immediately removed and submerged in fresh ice-cold Hanks' balanced salt solution (Gibco 14175-095). The dorsal cortices were cut into small pieces and dissociated into a single-cell suspension using a Papain Cell Dissociation Kit by following the manufacturer’s instruction (Miltenyi Biotec, catalog no. 130-092-628). The cells were subjected to fluorescence-activated cell sorting (FACS) to enrich for tdT^+^ cells. The Chromium droplet-based sequencing platform (10X Genomics) was used to generate scRNA-Seq libraries, following the manufacturer’s instructions (manual document part number: CG00052 Rev C). The cDNA libraries were purified, quantified using an Agilent 2100 Bioanalyzer, and sequenced on an Illumina Hiseq4000.

### scRNA-Seq analysis

High quality sequences (clean reads) were obtained by removing low quality sequences and joints. The clean reads were then processed with Cell Ranger software to obtain quantitative information on gene expression. Genes expressed in < 3 cells and cells with < 750 detected genes were filtered out. Cells with >10% mitochondrial genes were also filtered out. A global-scaling normalization method “Log Normalize” was applied to the raw read counts generated by 10X Cell Ranger to normalize the gene expression measurements for each cell by the total expression. The log-transformed normalized single-cell expression values were used for differential expression tests. Potential sources of variation, which may include technical noise, batch effects, and biological sources of variation such as cell-cycle stage, were regressed out to improve downstream dimensionality reduction and clustering. We regressed gene expression on the number of detected molecules per cell and the cell-cycle stage score. The scaled z-scored residuals were used for principal component analysis (PCA). Statistically significant principal components determined by a resampling test were kept for uniform manifold approximation and projection (UMAP) analysis. Differentially-expressed genes (DEGs) among clusters were identified by comparing cells in each cluster against all other cells with the likelihood-ratio test. Gene A was defined as a biomarker of cluster X if it was detected in ≥25% cells, and had an adjusted *P*-value <5%, and fold change ≥2 between cells of cluster X and all other cells. All these analyses were performed in the Seurat package v3.2 (https://satijalab.org/seurat/).

For analysis of cell lineages trajectory, we used a cell lineage inference algorithm, Slingshot (version 1.2.0, https://bioconductor.org/packages/slingshot/), to predict lineage trajectories and bifurcations by ordering cells along trajectories. Slingshot takes as input a matrix of reduced dimension normalized expression measures using PCA and cell clustering assignments. Lineages are defined by ordered sets of clusters beginning with the root node and terminating in the most distal cluster(s) with only one connection. Potential fitting curves are drawn to the subsets of cells that potentially make up each lineage. The ordering provided by Slingshot, analogous to pseudo-developmental time points, is referred to here as developmental order. The cluster representing radial glial cells (RGCs) was chosen as the starting root node. To analyze the lineage trajectory of cortical radial glial development, we extracted cells from the original cell dataset without PyN-related clusters. The most variable genes among all single cells were identified by Seurat. A pseudo-developmental timeline of single cells was calculated with the Slingshot package, using the most variable genes as time-ordering genes.

### scATAC-Seq analysis

Nuclei were isolated and washed according to the method supplied by the 10X Genomics: Nuclei Isolation for Single Cell ATAC Sequencing (CG000169). The isolated nuclei were re-suspended in chilled Diluted Nuclei Buffer (10X Genomics; 2000153) at a volume based on the number of starting cells and the final target concentration pf nuclei. Countstar (Rigel S2) was used to count the nuclei, and they were then immediately used to generate single-cell ATAC-seq libraries.

Following the 10X Genomics single-cell ATAC solution, by using the Chromium Chip E Single Cell Kit (Product Code 1000156) and Chromium Single Cell ATAC Library & Gel Bead Kit (Product Code 1000110), the nuclei in the bulk sample were partitioned into nanoliter-scale gel beads-in-emulsion, a pool of ~750,000 10 × barcodes was sampled to separately and uniquely index the transposed DNA of each individual nucleus, and libraries were generated (by CapitalBio Technology, Beijing). The libraries were sequenced using an Illumina Nova-seq sequencer with a sequencing depth of at least 25k read pairs per nucleus with a pair-end 50-bp reading strategy. Cell Ranger-ATAC pipeline Cell Ranger ATAC -1.2.0 and the mm10 reference genome were downloaded from the 10X Genomics website (https://support.10xgenomics.com/single-cell-atac/software/downloads/latest). Raw sequencing data were converted to fastq format using cell ranger-atac mkfastq. Cells with pct_reads_in_peaks >40, peak_region_fragments >3,000 and <80,000, TSS.enrichment >2.5, blacklist_ratio <0.01, and nucleosome_signal <4 were also filtered out. Peak calling, peak annotation, clustering visualization, TF motif enrichment analysis, and differential accessibility analysis were performed with the Signac package (https://satijalab.org/signac/index.html). Dimensionality was reduced using LSI, the top 2–30 principal components were used to generate clusters by calculating k-nearest neighbors and constructing the SNN graph and visualized *via* UMAP.

### Quantification and Statistical Analysis

scRNA-seq and scATAC-seq data analysis are described above. Statistical tests were performed using GraphPad Prism software, Microsoft Excel, and R. No statistical methods were used to estimate sample size. Numbers and percentages of cells are shown as the mean ± SEM.

### Data availability

scRNA-Seq and scATAC-Seq data have been deposited at the National Center for Biotechnology Information BioProjects Gene Expression Omnibus and are accessible through GEO Series accession number GSE161132.

## Results

### scRNA-Seq Analysis Reveals Cortical RGC Lineage at the Late Embryonic Stage

**Fig. 1:**
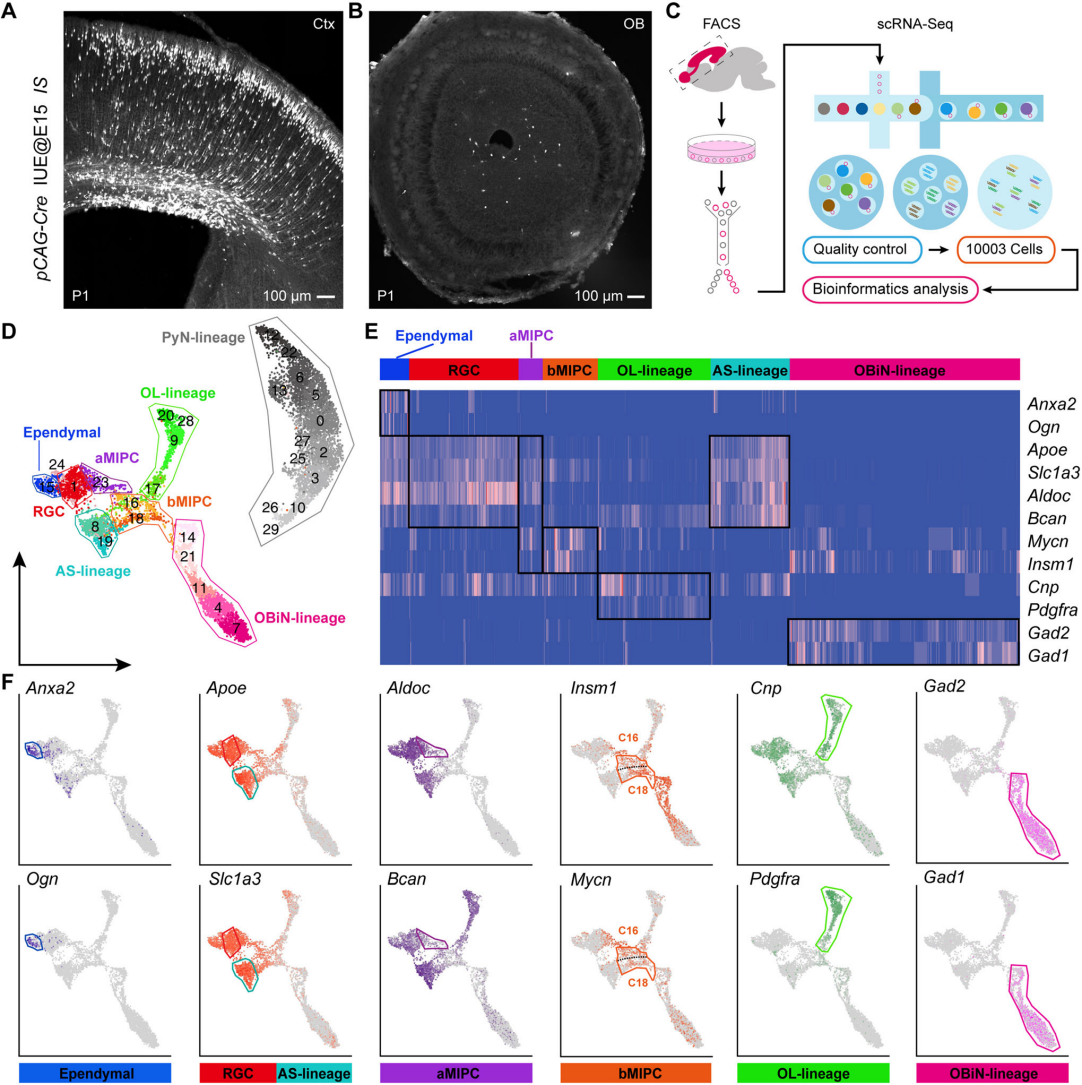
scRNA-Seq analysis of the P1 progeny of E15 cortical RGCs. **A, B** Representative images showing many cortical tdT^+^ cells and few OB tdT^+^ cells at P1 (*pCAG-Cre* plasmids were delivered into the cortical VZ of *IS* reporter mouse embryos at E15.0 by *in utero* electroporation). **C** Schematic of the workflow of scRNA-Seq analysis. Single tdT^+^ cells were isolated using FACS. **D** UMAP showing 30 clusters (C0–C29); the major cell types are annotated. **E** Heatmap showing cluster annotations, cell type assignments (x-axis) and expression of 12 marker genes (y-axis). **F** Feature plots of the 12 marker genes in 7 different cell types.

To characterize the cortical RGC lineage at the late embryonic stage, we delivered *pCAG-Cre* plasmids into the cortical VZ of *IS* reporter mouse embryos (*Rosa-CAG-LSL-Frt-tdTomato-Frt-EGFP*) [14] at E15.0 by IUE, labeling E15.0 cortical RGCs and their progeny. At P1, we found many tdTomato (tdT)^+^ cells in the cortical VZ, SVZ, and cortex, as well as a few tdT^+^ cells in the OB (Fig. 1A, B). Next, the IUE cortex, rostral migratory stream, and OB were dissected and dissociated into single-cell suspensions, and FACS was used to obtain individual tdT^+^ cells (Fig. 1C). We performed scRNA-Seq analysis on these FACS sorted tdT^+^ cells using the 10X Genomics platform. After removal of contaminating cells (microglia, endothelial cells, low-quality cells, outlier cells, and possible doublets), a total of 10,003 cells from 9 pups of 2 litters were recovered with a median of ~2,500 transcribed genes per cell. We performed regression analysis on these cells to remove the influence of cell cycle-dependent genes on cell-type identification.

The dimensionality of the data was reduced by unsupervised clustering using Louvian community detection. UMAP was then performed on these cells using Seurat 3.2 [17], which resulted in 30 clusters (C0–C29). Most of the clusters overlapped with adjacent clusters, suggesting highly-related transcriptomes (Fig. 1D). To more precisely define the identities to the 30 clusters we used marker genes; this resulted in 8 discrete populations: ependymal cells, RGCs, aMIPCs, bMIPCs, PyN-lineage, OL-lineage, AS-lineage, and OBiN-lineage. These clearly segregated clusters exhibited distinct molecular signatures (Fig. 1D, E). We hypothesize that these clusters are all lineally related to cortical RGCs, and our nomenclature from here on will reflect this. Below, we describe the molecular features of these 8 putative cell lineages.

PyN-lineage contained 13 clusters, 48% of cells (4,772/10,003), including PyN-IPCs and PyNs (Fig. S1A-D). In PyN-IPCs (C26 and C29), enriched genes included *Pax6, Neurog2, Ascl1, Eomes (Tbr2), Insm1, Notch1,* and *Neurod1*, as well as the cell proliferation markers *Mki67, Top2a*, and *Cdk1* (Fig. S1A). Expression of PyN common marker genes *Bcl11a, Neurod2, Neurod6, Emx1* and *Lhx2* was also observed (Fig. S1B). Because RGCs were labeled at E15.0, only a few deep layer PyNs (*Bcl11b*^+^, *Fezf2*^+^, and *Sox5*^+^) were generated (Fig. S1C). In contrast, most upper layer PyNs expressed *Bhlhe22, Cux1, Cux2, Pou3f1, Tbr1*, and *Satb2* (Fig. S1D).

Terminally differentiated ependymal cells are directly converted from RGCs at late embryonic stages, and form motile multicilia during the first postnatal week [5]. C15 consisted of immature ependymal cells, which expressed *Anxa2, Ogn, Cryab, Enkur, Foxj1, Itih5, Lgals3, Prom1 (CD133), S100a11,* and *Tspan15* (Figs 1F, S1E), typical markers for ependymal cells [18].

**Fig. 2:**
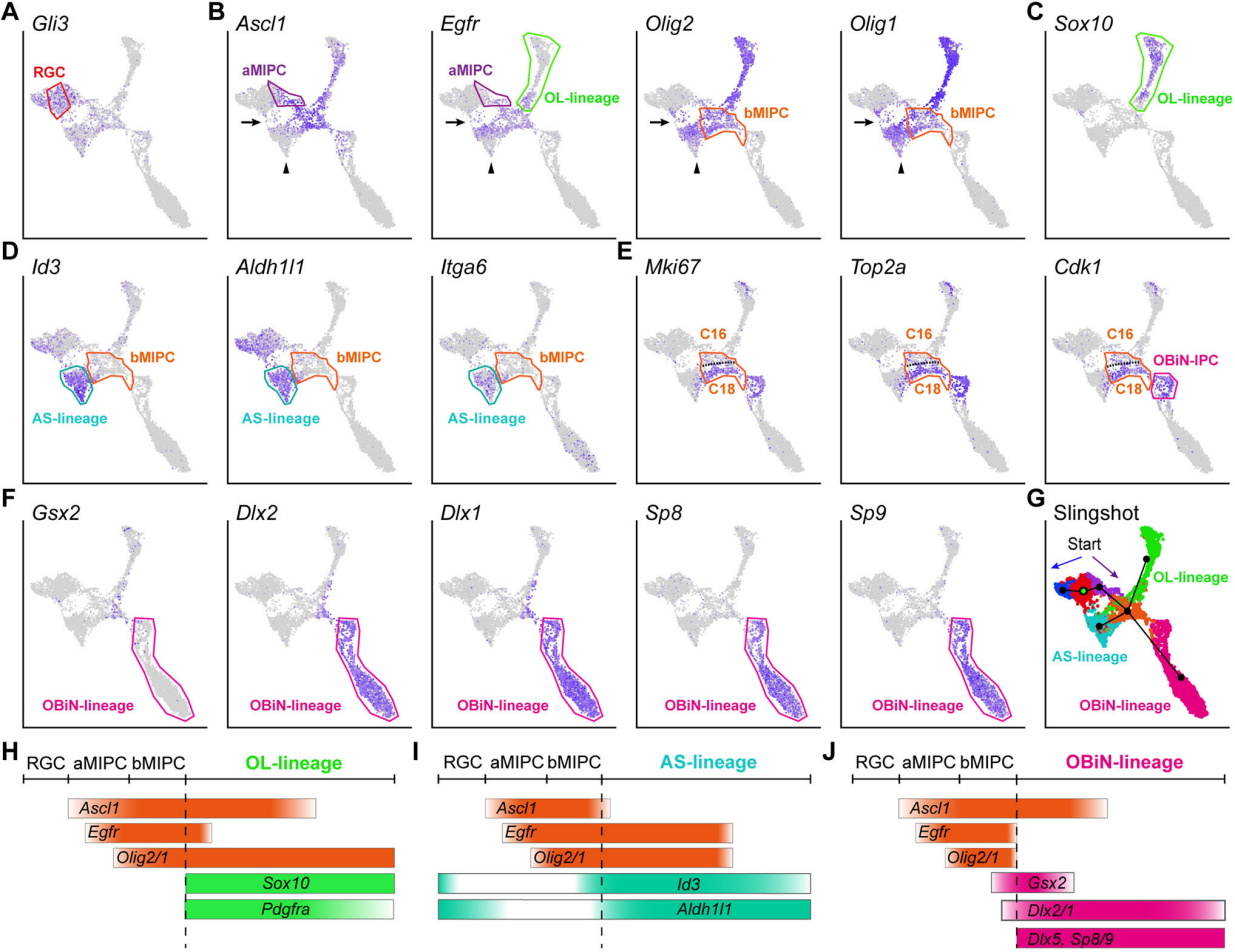
Expression of IPC-specific marker genes and their lineage progression. **A-F** Feature plots of marker genes for RGCs, aMIPCs, bMIPCs, and OL-lineage, AS-lineage, OBiN-lineage cells. Broad cell types (cell lineages) are shown rather than individual clusters. Note a small number of cells between RGCs and AS-lineage cells (arrows in **B**); they might be RGCs that were translocating to the cortex. Also note the gradual downregulation of *Ascl1, Egfr*, *and Olig2/1* expression in AS-Iineage cells (arrowheads in **B**). **G** Predicted cell lineage trajectories from RGCs. **H-J** Schematic of the aMIPC and bMIPC lineage progression.

In the P1 cortex, cortical RGCs (C1 and C24) and AS-lineage cells (C8 and C19) expressed both shared and distinct genes. *Fezf2, Emx1, Hopx, Gfap, Naaa*, *Nkain4*, and *Gli3* were mainly expressed in RGCs but not the AS-lineage (Fig. 2A, Fig. S1F). On the other hand, *Egfr, Olig2, Olig1, Id3, Itga6, Fgfr3, Grm3*, and *Grm5* were mainly expressed in the AS-lineage, but not RGCs (Figs 2B, D and S1G). There were many genes expressed by both RGCs and AS-lineage cells at P1. Examples of such genes included *Aldh1l1, Aldoc, Apoe, Aqp4, Bcan, Dbi, Fabp7, Gja1, Glul, Mfge8, Ncan, Nes, Slc1a2, Scl1a3, Sox1, Sox2, Sox9, Tnc, Vim, Zfp36l1, Hes1, Hes5, Hey1*, and *Notch1* (Figs 1F, 2D, S1H, S2H). Most AS-lineage cells in the P1 cortex were immature as they did not express *S100b* (Fig. S1G). A subpopulation of these cells were dividing, expressing *Mki67*, *Top2a*, and *Cdk1* (Fig. 2E) [19].

Oligodendrocytes in the P1 cortex were also immature, as they did not express *Mbp* and *Mog* (Fig. S2A). Thus, the OL-lineage included both OL-lineage-restricted progenitor cells (also known as oligodendrocyte progenitor cells, OPCs) and immature oligodendrocytes. OL-lineage expressed *Egfr, Ascl1, Olig2/1, Pdgfra, Sox10, Cnp, C1ql1, Chd7, Chd8, Dll1, Dll3, Cspg4 (Ng2), Pcdh15*, and *S100b* (Figs 1F, 2B, C, S1G, S2A). Similarly, the OBiN-lineage included both OBiN lineage-restricted progenitor cells (OBiN-IPCs) and immature OBiNs. Consistent with our previous results, cells from OBiN-IPCs to immature OBiNs specifically expressed the gene regulatory network *Gsx2* – *Dlx1/2/5/6* – *Sp8/Sp9* – *Tshz1*– *Prokr2* (Figs 2F, S2B), which is critical for the generation of nearly all OBiNs [20–24]. In addition, OBiN-lineage cells also expressed *Arx, Etv1, Gad2/1, Pax6,* and *Pbx3,* as well as the cell proliferation markers *Mki67, Top2a*, and *Cdk1* (Figs 1F, 2E, S2B). Very few cortex-derived OBiNs expressed *Htr3a* (Fig. S2B).

We also found that many genes were widely expressed by progeny of cortical RGCs at P1 (nearly all clusters), such as *Foxg1, Meis2, Nfia, Nfib, Nifx, Ppp1r14b, Tcf4, Smarca4*, and *Zbtb20* (Fig. S2C). Taken together, using IUE to label E15.0 cortical RGCs and their progeny, combined with scRNA-Seq analysis, we have identified the principal P1 progeny of E15.0 cortical RGCs.

### Transcriptional Profiles of Cortical aMIPCs and bMIPCs, and Their Lineage Progression

A key feature of E15.0 cortical RGCs is their expression of *Gli3* (Fig. 2A). Previously, we demonstrated that cortical RGCs have increased sonic hedgehog (SHH) signaling at the late embryonic stages which results in reduced GLI3R protein, and the generation of cortical oligodendrocytes, astrocytes, and OBiNs [13]. Based on the expression of specific genes across several neighboring clusters, we were able to deduce their relationships, and establish continuities between specific clusters and developmental lineages. For example, RGCs strongly expressed *Apoe*, *Slc1a3*, *Aldoc*, and *Bcan*, and aMIPCs continued to express these genes, albeit at lower levels (Fig. 1F), providing evidence that these aMIPCs are derived from RGCs.

Compared with RGCs, the most prominent feature of aMIPCs (C23) was the increase in expression of *Ascl1, Egfr* (Fig. 2B), *Insm1*, and *Mycn* (Fig. 1F). *Ascl1* is a proneural transcription factor, playing a pivotal role in promoting cell proliferation and differentiation [25]. However, most aMIPCs did not express the canonical cell proliferation markers *Mki67, Top2a*, and *Cdk1* (Fig. 2E), suggesting that they are “immature” or quiescent. By contrast, C16 and C18 bMIPCs were “mature” IPCs, as they expressed higher levels of *Ascl1* and *Egfr,* as well as the cell proliferation genes *Mki67*, *Top2a*, and *Cdk1*, than aMIPCs (Fig. 2B, E). Most importantly, they expressed the *Olig2* and *Olig1* transcription factors (Fig. 2B). It is worth noting that C16 and C18 bMIPCs shared a large number of identifying markers but formed separate clusters. Indeed, compared with C16, C18 expressed even higher levels of the cell proliferation markers *Mki67, Top2a, Cdk1, Birc5, Ccnb2, Pbk, Tpx2,* and *Ube2c* (Figs 2E, S2D), the histone genes *Hist1h1b, Hist1h1e, Hist1h2ae,* and *Hist1h2ap* (Fig. S2E), and the centromere protein genes *Incenp, Cenph, Cenpl, Cenpm, Cenpn, Cenpq,* and *Cenpw* (Fig. S2F). This suggests that C18 has higher proliferative activity than C16.

Some bMIPCs started to express the AS-IPC marker genes *Id3* and *Aldh1l1* (Fig. 2D) [26, 27] and the OBiN-IPC marker genes *Gsx2* and *Dlx2/1* [20] (Fig. 2F, J). Although different levels of *Id3* and *Aldh1l1* were expressed by RGCs in late embryogenesis, their expression was largely downregulated in aMIPCs (Fig. 2D, I). Thus, the re-emergence and uneven expression of many lineage-restricted genes in bMIPCs indicates they were heterogeneous. The heterogeneity of bMIPCs suggests their progression toward different developmental trajectories. Slingshot analysis [28] predicted a developmental trajectory and pseudo-timeline progression of the progenitor clusters. The lineage progression was predicted to start from RGCs passing through aMIPCs, after which dividing bMIPCs were identified (Fig. 2G). The aMIPC population, upregulating *Ascl1* and *Egfr* prior to typical cell-cycle genes, and the bMIPC population, further upregulating *Ascl1, Egfr, Olig2/1, Mki67, Top2a*, and *Cdk1*, were located between RGCs and lineage-restricted IPCs, representing transitional cell types (Fig. 2G).

*Sox10* is expressed in OPCs and mature oligodendrocytes [29]. *Ascl1*^*+*^*Egfr*^*+*^*Olig2/1*^+^ bMIPCs first gave rise to *Ascl1*^*+*^*Egfr*^*+*^*Olig2/1*^*+*^*Pdgfra*^*+*^*Sox10*^*+*^ OPCs. OPCs maintained *Ascl1*, *Olig2/1, Pdgfra*, and *Sox10* expression but soon downregulated *Egfr* expression (Fig. 2C, H). *Ascl1*^*+*^*Egfr*^*+*^*Olig2/1*^*+*^ bMIPCs also gave rise to *Egfr*^*+*^*Olig2/1*^*+*^*Id3*^*+*^ AS-IPCs that downregulated *Ascl1* expression (Fig. 2I). Maturing astrocytes further downregulated *Olig2/1* and *Egfr* expression (arrowheads in Fig. 2B), but expressed *Id3, Aldh1l1*, and *Itga6* (Fig. 2D, I) [26, 30, 31]. When *Gsx2* and *Dlx2/1* expression was upregulated in the bMIPCs, *Egfr* and *Olig2/1* were downregulated first, followed by *Ascl1* (Fig. 2J). *Dlx1/2* further induced *Dlx5/6* and *Sp8/9* expression [20, 32]; these dividing *Dlx1/2/5/6*^*+*^*Sp8/9*^*+*^ cells were identified as OBiN-IPCs (Fig. 2E, F).

In addition, we found a small number of cells located between the RGCs and AS-lineage cells. Interestingly, these cells also expressed *Ascl1*, *Egfr*, and *Olig2/1* (arrows in Fig. 2B) which might represent those RGCs that were translocating to the cortex and transforming into AS-IPCs. Taken together, our scRNA-Seq analysis revealed that neocortical RGCs in late embryogenesis first generated aMIPCs, which quickly differentiated into bMIPCs, and most OPCs, AS-IPCs, and OBiN-IPCs went through this bMIPC state before their cell fates were committed. In other words, the bMIPC population gave rise to most of the cortical oligodendrocytes and astrocytes, and a subpopulation of OBiNs.

### *In Vivo* Validation of Markers of Different Cell Types in the Developing Cortex

**Fig. 3:**
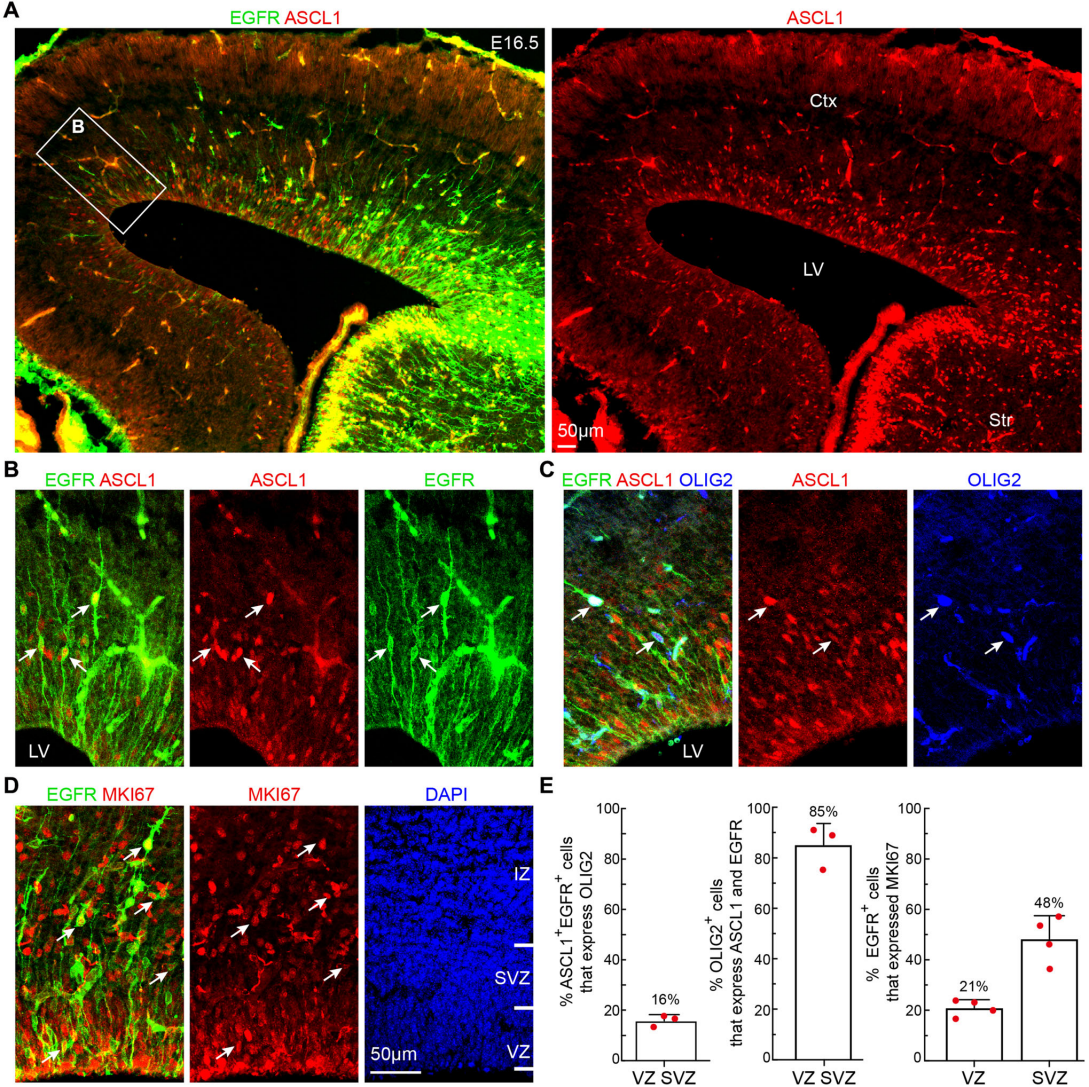
Identification of aMIPCs and bMIPCs in E16.5 cortex. **A** ASCL1 and EGFR expression in the cortex at E16.5. Note that both ASCL1 and EGFR are expressed in a high lateral (ventral) to low medial (dorsal) gradient in the cortex. **B** Higher magnification of the boxed area in **A** showing bipolar ASCL1^+^EGFR^+^ aMIPCs (arrows). **C** OLIG2 expression in ASCL1^+^EGFR^+^ cells (arrows). Non-specific binding of OLIG2 mouse monoclonal antibody to E16.5 brain sections is evident. **D** EGFR^+^MKI67^+^ cells in the cortex (arrows). **E** Quantification of the above immunostaining experiments. Mean ± SEM (*n* = 3–4 mice). Ctx, cortex; IZ, intermediate zone; LV, lateral ventricle; Str, striatum.

To validate the molecular signatures of the diverse cortical cell types identified from our scRNA-Seq data, we investigated the expression of cell subtype specific marker proteins beginning at E16.5, using double- or triple-immunofluorescence analysis and confocal microscopy. First, we examined ASCL1, EGFR, and OLIG2 expression in the cortex. ASCL1 is known to be expressed at low levels in PyN-IPCs [33]. At E16.5, the time when the production of PyNs ceases, ASCL1 and EGFR were expressed in a lateral (ventral) high to medial (dorsal) low gradient in the cortical VZ/SVZ (Fig. 3A). Most importantly, although ASCL1 and EGFR were strongly expressed in the ganglionic eminences (GEs) of the ventral telencephalon, their expression was down-regulated when GE-derived cells migrated into the cortex (Fig. 3A). Thus, ASCL1^+^ and EGFR^+^ cells that were observed in the cortex were generated from the cortex itself. EGFR is a cell-surface tyrosine kinase receptor belonging to the *ErbB* family of receptors. There were not many EGFR^+^ cells in the medial cortex at E16.5, as EGFR expression had just begun, providing an opportunity to examine their morphology. In the cortical VZ, we found that 70% of EGFR^+^ cells were bipolar, possessing an apical (ventricular) end-foot and a basal process of variable length (Fig. 3B). We did not observe EGFR^+^ cells that exhibited bipolar morphology with one end-foot on the ventricular surface and an elongated radial process that projected toward the pial surface, suggesting that they are not RGCs. We have termed these EGFR^+^ cells aMIPCs as they are multipotent and able to generate both cortical glia and OBiNs. During the period of PyN neurogenesis, this cell type is called short neural precursors (also known as apical intermediate progenitors) [34, 35], which are unipotent and exclusively generate PyNs.

At E16.5, in addition to the ventral GE-derived OLIG2^+^ cells in the cortex, a very small number of OLIG2^+^ cells were observed in the cortical VZ and SVZ, 85% of which were ASCL1^+^EGFR^+^, while only 16% of ASCL1^+^EGFR^+^ cells expressed OLIG2 (Fig. 3C, E). This suggests that cortical RGCs first generate ASCL1^+^EGFR^+^ cells, which in turn differentiate into ASCL1^+^EGFR^+^OLIG2^+^ cells. In the cortical VZ, 21% of EGFR^+^ cells expressed MKi67. In contrast, 48% of EGFR^+^ cells expressed MKi67 in the SVZ, most of which were multipolar (Fig. 3D, E).

**Fig. 4:**
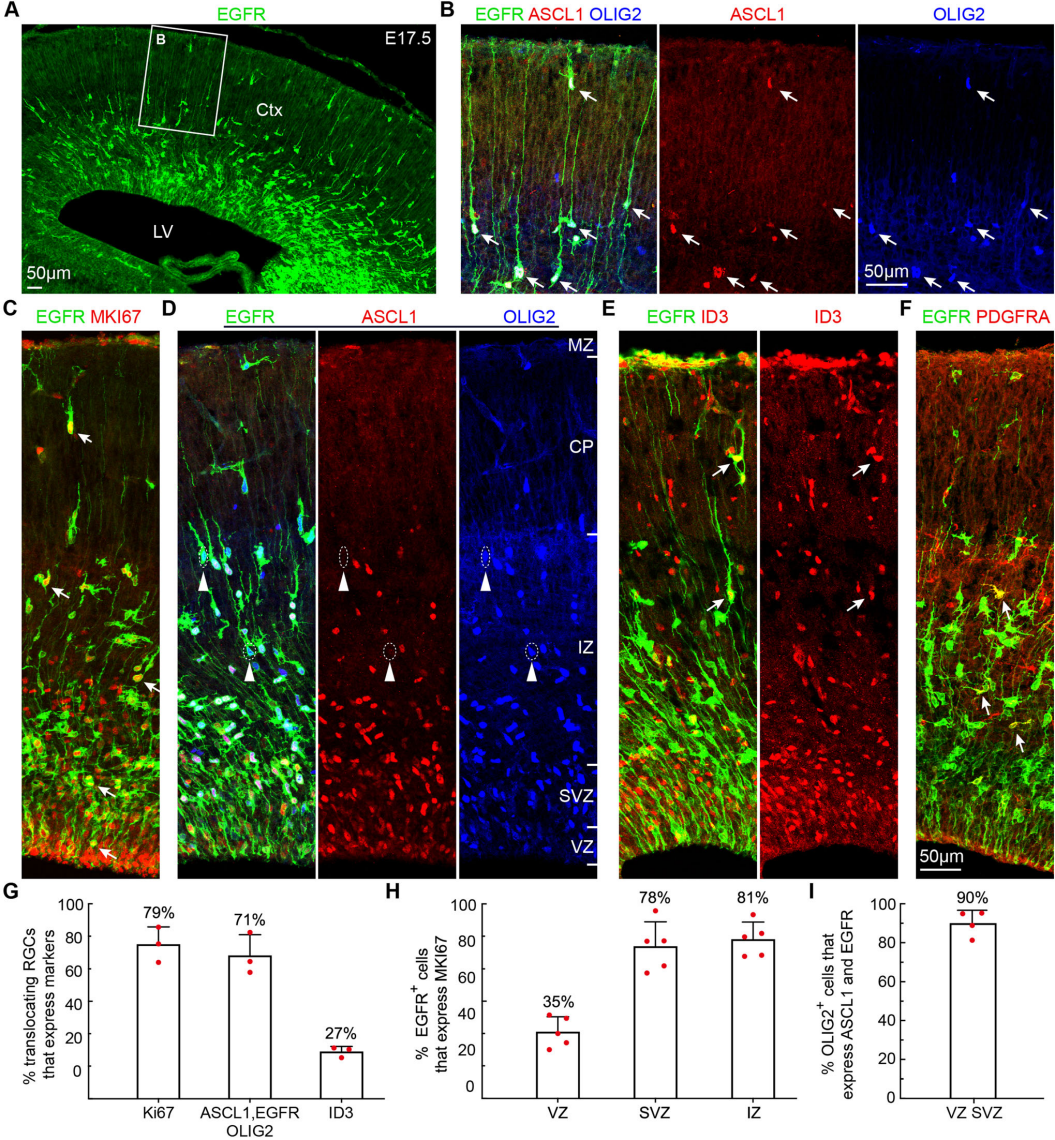
*In Vivo* validation of markers for aMIPCs, bMIPCs, AS-IPCs and OPCs in the E17.5 cortex. **A** EGFR is expressed in a high lateral (ventral) to low medial (dorsal) gradient in the E17.5 cortex. **B** Most translocating RGCs express EGFR, ASCL1, and OLIG2 (arrows). **C** EGFR^+^MKI67^+^ cells (arrows). **D** EGFR, ASCL1, and OLIG2 triple immunostained cortical section. Note a few translocating RGCs with downregulated ASCL1 expression (arrowheads). **E** A few translocating RGCs (AS-IPCs) express ID3 (arrows). **F** EGFR^+^PDGFRA^+^ OPCs in the cortex (arrows). **G-I** Quantification of the above immunostaining experiments. Mean ± SEM (*n* = 3–5 mice). CP, cortical plate; Ctx, cortex; IZ, intermediate zone; LV, lateral cortex; MZ, marginal zone.

At E17.5, a subpopulation of RGCs was translocating to the cortical plate (Fig. 4A, B), suggesting that they are transforming into AS-IPCs [2–4]. Based on their morphology, we observed that 79% of translocating RGCs expressed MKI67 (Fig. 4C, G), of which 71% expressed ASCL1, EGFR, and OLIG2 and 29% expressed EGFR and OLIG2, but ASCL1 was downregulated (Fig. 4B, D, G). Meanwhile, we found that 27% of translocating RGCs expressed ID3 (Fig. 4E, G). Thus, translocating RGCs that express EGFR and ID3 are the earliest AS-IPCs in the cortex.

OPCs that expressed EGFR and PDGFRA were also found in the E17.5 cortex (Fig. 4F), consistent with evidence that cortical oligodendrogenesis begins around E17.5 [36]. In the E17.5 presumptive somatosensory cortex, we found that 35% of EGFR^+^ cells in the VZ expressed MKI67, while 78% of EGFR^+^ cells in the SVZ and 81% of EGFR^+^ cells in the IZ expressed MKI67 (Fig. 4C, G), indicating their higher proliferative activity. In the VZ/SVZ, 90% of OLIG2^+^ cells expressed ASCL1 and EGFR (Fig. 4D, G). In the IZ and cortical plate, all EGFR+ cells co-expressed OLIG2 (Fig. 4D); these EGFR^+^OLIG2^+^ cells were either OPCs (EGFR^+^OLIG2^+^PDGFRA^+^) or AS-IPCs (EGFR^+^OLIG2^+^ID3^+^).

**Fig. 5:**
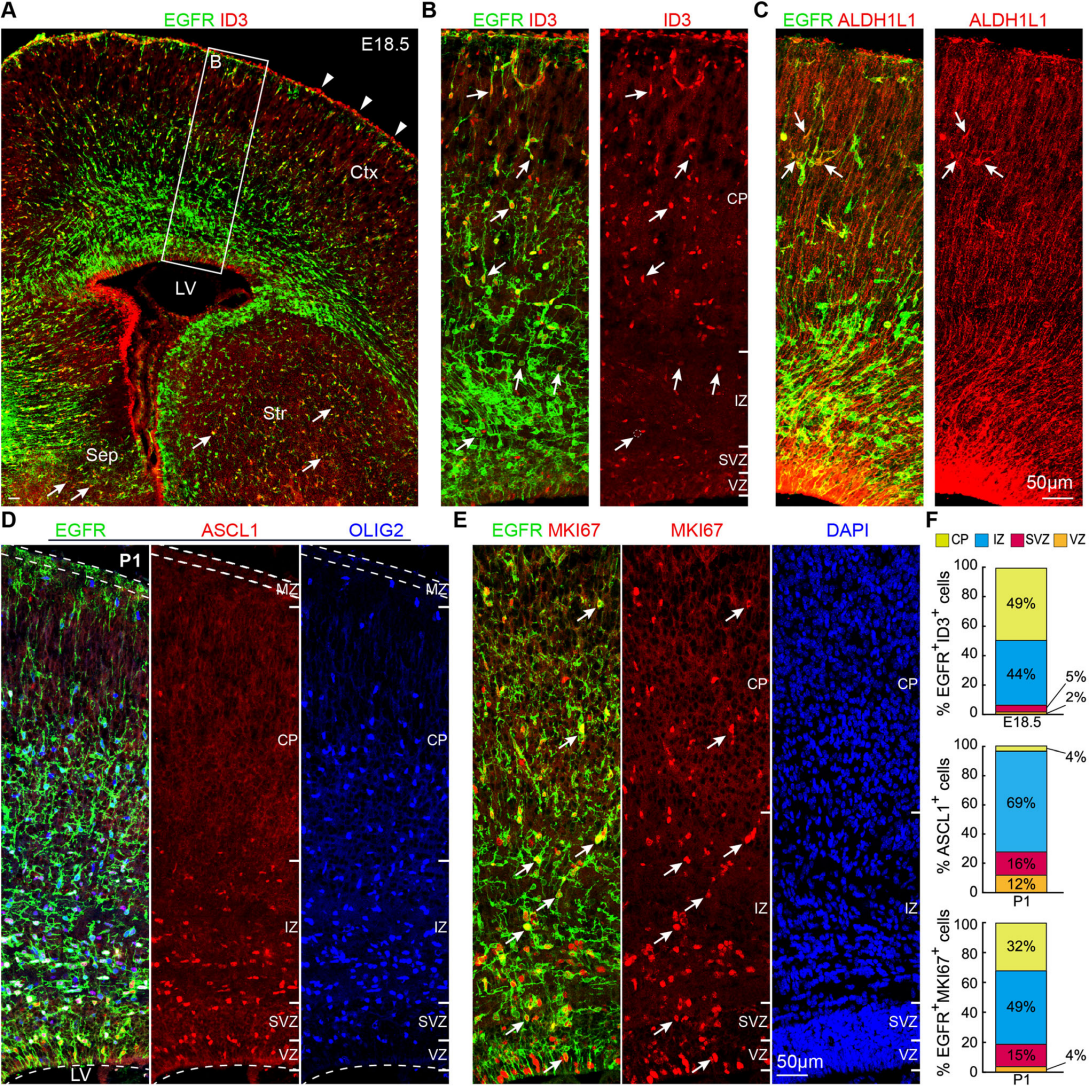
Identification of AS-IPCs in the E18.5 cortex. **A, B** EGFR^+^ and/or ID3^+^ cells in the cortex (Ctx) with higher magnification images at E18.5. EGFR^+^ID3^+^ AS-IPCs (arrows) are evident in the SVZ, intermediate zone (IZ), and cortical plate (CP). Note ID3 expression in the blood vessels of the meninges (arrowheads). EGFR^+^ID3^+^ AS-IPCs (arrows) also occur in the septum (Sep) and striatum (Str). **C** EGFR^+^ALDH1L1^+^ AS-IPCs (arrows) mainly occur in the cortical plate. **D, E** Expression of EGFR, ASCL1, OLIG2, and MKI67 in the somatosensory cortex at P1. Note that most EGFR^+^ cells in the VZ contact the lateral ventricle. MZ, marginal zone. **F** Quantification of the laminar distribution of EGFR^+^ID3^+^ in the E18.5 cortex, ASCL1^+^ cells and EGFR^+^MKI67^+^ cells in the P1 cortex. Means ± SEM (*n* = 3 mice).

At E18.5, in addition to the cortical plate, EGFR^+^ID3^+^ AS-IPCs were observed in the SVZ/IZ (Fig. 5A, B, F); they were most likely derived from bMIPCs and not from translocating RGCs. EGFR^+^ID3^+^ AS-IPCs were also found in the septum and striatum (Fig. 5A). Although ID3 expression in the AS-lineage was earlier than that of ALDH1L1, we were able to find a very small number of EGFR^+^ALDH1L1^+^ AS-IPCs in the E18.5 cortex (Fig. 5C). In the P1 cortical VZ and VZ/SVZ border, 76% of EGFR^+^ cells contacted the lateral ventricle (Fig. 5D, E). We observed that 96% of ASCL1^+^ cells were located in the VZ/SVZ/IZ (Fig. 5D, F), suggesting that most OPCs and AS-IPCs had downregulated ASCL1 expression in the cortical plate. The majority of EGFR^+^MKI67^+^ cells (81%) was in the IZ (white matter) and cortical plate (cortex). Only 4% of these cells were in the VZ and 15% in the SVZ (Fig. 5E, F). Taken together, analysis of IPC marker protein expression in the E16.5, E17.5, E18.5, and P1 cortex revealed that cortical RGCs first give rise to ASCL1^+^EGFR^+^ “immature” aMIPCs. aMIPCs then quickly differentiate into “mature” bMIPCs that express ASCL1, EGFR, OLIG2, and MKI67, which undergo several rounds of divisions to generate OPCs and AS-IPCs. OBiN-IPCs were also generated at E18.5 [13]. These immunostaining results are consistent with the scRNA-Seq developmental trajectory analysis (Fig. 2G).

Based on the numbers of SOX10^+^, EGFR^+^ID3^+^, and ALDH1L1^+^ cells in the cortical plate (cortex), we examined the development of cortical oligodendrocytes and astrocytes from E17.5 to P21 (Fig. S3A-C). ALDH1L1^+^ cells were first observed in the ventral cortex at E17.5 (Fig. S3A). Apart from those ventral GE-derived oligodendrocytes, cortex-derived SOX10^+^ cells were also first found in the ventral cortex (Fig. S3A). Thus, cortical astrocytes and oligodendrocytes first developed in the ventral cortex followed by those in the dorsal cortex, consistent with the developmental gradient of cortical PyNs [37]. EGFR^+^ID3^+^ cells were found earlier than ALDH1L1^+^ cells in the cortex, but from P1, the number of ALDH1L1^+^ cells gradually surpassed the number EGFR^+^ID3^+^ cells due to downregulation of EGFR expression (Fig. S3C). Thus, ALDH1L1 is an excellent pan-astrocytic marker in the postnatal brain [26].

### Lineage Tracing Reveals that Cortical ***Ascl1***^+^ Cells Generate Cortical Oligodendrocytes, Astrocytes, and OBiNs

**Fig. 6:**
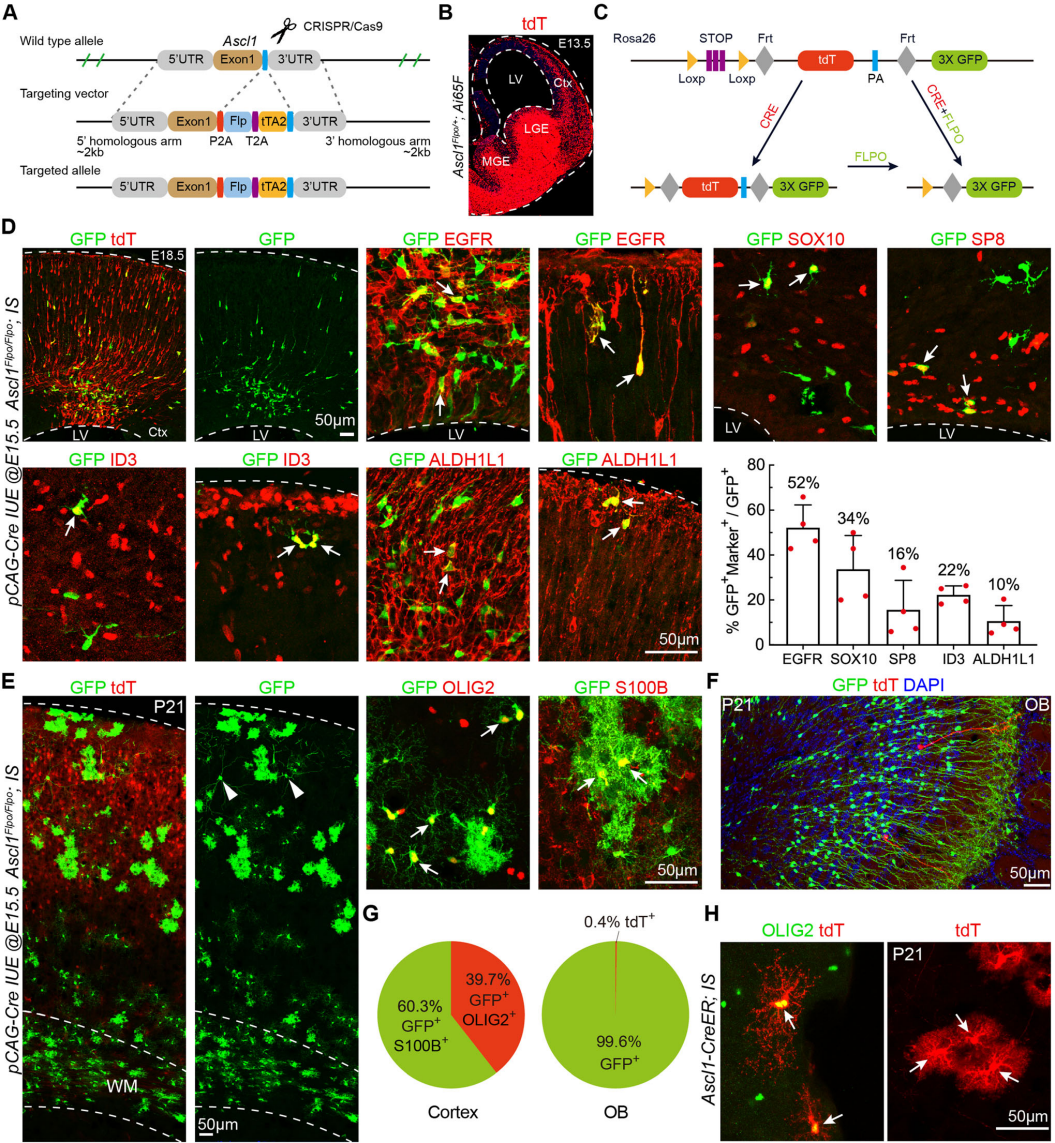
Cortical RGC-derived *Ascl1*^+^ MIPCs give rise to cortical oligodendrocytes, astrocytes, and OBiNs. **A** Strategy for generating the *Ascl1*^*Flpo*^ allele. **B** Representative image of tdT^+^ cells in the brain of an *Ascl1*^*Flpo/+*^*; Ai65F* (*Rosa26-tdT-FRT*) mouse at E13.5. **C** Strategy of intersectional lineage analysis. **D** Representative images and counts of cortical GFP^+^ cells that express EGFR, SOX10, SP8, ID3, and ALDH1L1 (*pCAG-Cre* plasmids were electroporated to the cortical VZ of *Ascl1*^*Flpo/Flpo*^*; IS* mice at E15.5 and the brains were analyzed at E18.5). Mean ± SEM (*n* = 4 mice). **E** Cortical GFP^+^ oligodendrocytes and GFP^+^ astrocytes (left) and at higher magnification (right) at P21. Note a few GFP^+^ PyNs in the cortical upper layers (arrowheads). **F** GFP^+^ and tdT^+^ interneurons in the OB at P21. **G** Percentages of GFP^+^OLIG2^+^ oligodendrocytes *vs* GFP^+^S100^+^ astrocytes (left) in the cortex, and percentages of GFP^+^ interneurons *vs* tdT^+^ interneurons in the OB at P21 (right) (*n* = 3 mice). **H** Images of tdT^+^ oligodendrocytes and tdT^+^ astrocytes (arrows) in the cortex (tamoxifen was injected into *Ascl1-CreER; IS* mice at E17.5 and the brains were analyzed at P21). Ctx, cortex; LGE, lateral ganglionic eminence; LV, lateral ventricle; MGE, medial ganglionic eminence; WM, white matter.

To investigate the cell lineages of *Ascl1*^+^ MIPCs, we generated the *Ascl1*^*Flpo*^ knockin allele by inserting a *P2A-Flpo-tTA2* cassette immediately before the stop codon of the endogenous *Ascl1* gene (Fig. 6A). We confirmed the specificity of *Flpo* activity in the *Ascl1*^+^ cells by breeding the *Ascl1*^*Flpo/+*^ mice with *Ai65F* (*Rosa26-tdTomato-FRT*) mice [14, 38]. The expression of tdT was observed in the GEs at E13.5 (Fig. 6B), consistent with the expression pattern of ASCL1. To specifically label cortical RGC-derived *Ascl1*^+^ cells, we performed intersectional analysis using *Cre* and *Flpo* recombinases in combination with *IS* reporter mice (Fig. 6C). To perform intersectional lineage analysis, we delivered *pCAG-Cre* plasmids specifically into the cortical VZ of *Ascl1*^*Flpo/Flpo*^; *IS* embryos at E15.5 by IUE. In this experiment, cells generated from electroporated cortical RGCs, that did not go through an *Ascl1*^+^ stage, expressed tdT. On the other hand, cells generated from the electroporated RGCs, that did go through an *Ascl1*^+^ stage, expressed GFP. We examined the cortex at E18.5. The electroporation sites were confirmed by examining tdT expression (Fig. 6D). We observed many tdT^+^ cells that extended from the IUE cortical VZ to the cortical plate. Many GFP^+^ cells were also seen in the cortical VZ, SVZ, IZ, and cortical plate (Fig. 6D). We did not observe GFP^+^ cells that had bipolar morphology with one end-foot on the ventricular surface and an elongated radial process that projected toward the pial surface (Fig. 6D), further providing evidence that *Ascl1*^+^ cells in the cortex were not RGCs. Among the GFP^+^ cells, 52% expressed EGFR, 34% expressed SOX10, 16% expressed SP8, 22% expressed ID3, and 10% expressed ALDH1L1 (Fig. 6D).

We next examined the progeny of cortical *Ascl1*^+^ cells in the P21 brain. In the neocortex, tdT^+^ cells were mainly upper layer PyNs (Fig. 6E). Many GFP^+^ cells were found and their morphology and expression of OLIG2 and S100B indicated that they were oligodendrocytes or astrocytes (Fig. 6E). The ratio of astrocytes to oligodendrocytes was 1.5 :1 (Fig. 6G). A few cortical GFP^+^ PyNs were also observed (Fig. 6E) as relatively low levels of ASCL1 are expressed in PyN-IPCs [33]. In the OBs, a large number of GFP^+^ OBiNs but very few tdT^+^ OBiNs were found (Fig. 6F, G), indicating that virtually all cortex-derived OBiNs go through an *Ascl1*^+^ MIPC stage.

To confirm the above results from *Ascl1-Flpo* lines, we next used *Ascl1-CreER* knockin knockout mice [39] to determine whether *Ascl1*^+^ cells generate cortical astrocytes. Astrocytes are allocated to spatial domains in the brain in accordance with their embryonic sites of origin in the VZ [31, 40]. Thus, cortical astrocytes are derived from the cortex itself. We fate-mapped *Ascl1*^+^ IPCs by transiently activating Ascl1-CreER in *Ascl1-CreER; IS* mice by tamoxifen injection at E17.5, followed by immunohistochemical analysis at P21. As expected, many tdT^+^ astrocytes and oligodendrocytes were found in the cortex (Fig. 6H). Taken together, our results demonstrated that cortical *Ascl1*^+^ MIPCs at late embryonic stages give rise not only to cortical oligodendrocytes and astrocytes but also to OBiNs.

### ***Olig2***^+^ Cells Generate Nearly All Cortical Oligodendrocytes and Astrocytes, and a Subpopulation of OBiNs

**Fig. 7:**
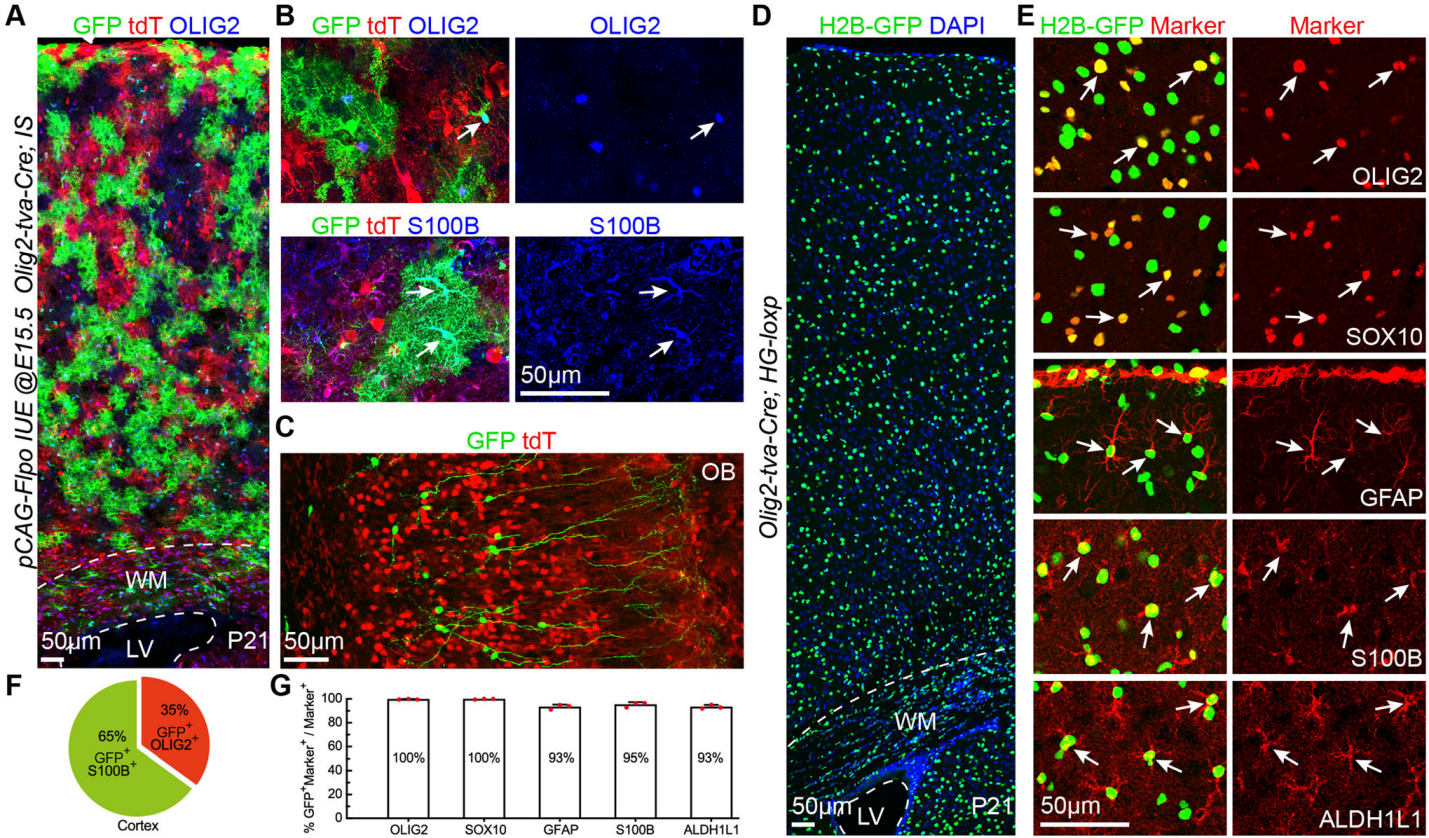
Cortical RGC-derived *Olig2*^+^ bMIPCs give rise to the vast majority of cortical oligodendrocytes and astrocytes, and a subpopulation of OBiNs. **A** Representative images showing GFP^+^ cells derived from cortical RGCs (*pCAG-Flpo* plasmids were electroporated to the cortical VZ of *Olig2-tva-Cre*; *IS* mice at E15.5 and the brains were analyzed at P21). **B** Higher magnification image showing GFP^+^OLIG2^+^ (arrow) and GFP^+^S100B^+^ cells (arrows) in the cortex. **C** GFP^+^ interneurons and tdT^+^ cells in the OB. **D** H2B-GFP^+^ cells in the P21 cortex of an *Olig2-tva-Cre; HG loxp* mouse. **E** Double immunofluorescence labeling for H2B-GFP and markers (arrows) for cortical oligodendrocytes (OLIG2 and SOX10) and astrocytes (GFAP, S100B, and ALDH1L1). **F** Percentages of GFP^+^S100B^+^ astrocytes *vs* GFP^+^OLIG2^+^ oligodendrocytes in the cortex (*n* = 3 mice). **G** Histogram summarizing the percentages of H2B-GFP^+^ cells that express different markers (*n* = 3 mice).

We next examined the lineage of cortical *Olig2*^+^ bMIPCs. We delivered *pCAG-Flpo* plasmids into the cortical VZ of E15.5 *Olig2-tva-Cre*; *IS* embryos [15] by IUE and brains were analyzed at P21. In this experiment, all cells that were generated from cortical and subcortical *Olig2*^+^ cells expressed tdT. On the other hand, cells generated from the electroporated cortical RGCs that went through an *Olig2*^+^ stage expressed GFP. From the SVZ to the cortex at P21, we found a large number of GFP^+^ cells that expressed either OLIG2 or S100B, indicating that they were oligodendrocytes (35%) or astrocytes (65%) (Fig. 7A, B, F). GFP^+^ OBiNs were also found (Fig. 7C). We also crossed *Olig2-tva-Cre* lines with Cre dependent H2B-GFP reporters (HG-loxp mice) [14] and examined the identities of H2B-GFP^+^ cells in the P21 cortex (Fig. 7D). Nearly all cortical OLIG2^+^, SOX10^+^, GFAP^+^, S100B^+^, and ALDH1L1^+^ cells were labeled by H2B-GFP (Fig. 7E, G). This experiment demonstrated that all cortical oligodendrocytes and astrocytes are derived from *Olig2*^*+*^ cells, including the minority of cortical oligodendrocytes that originated from the GE of the ventral telencephalon.

### scATAC-Seq Analysis Confirms the Gene Regulatory Logic Underlying Cortical Glia and OBiN Differentiation

**Fig. 8:**
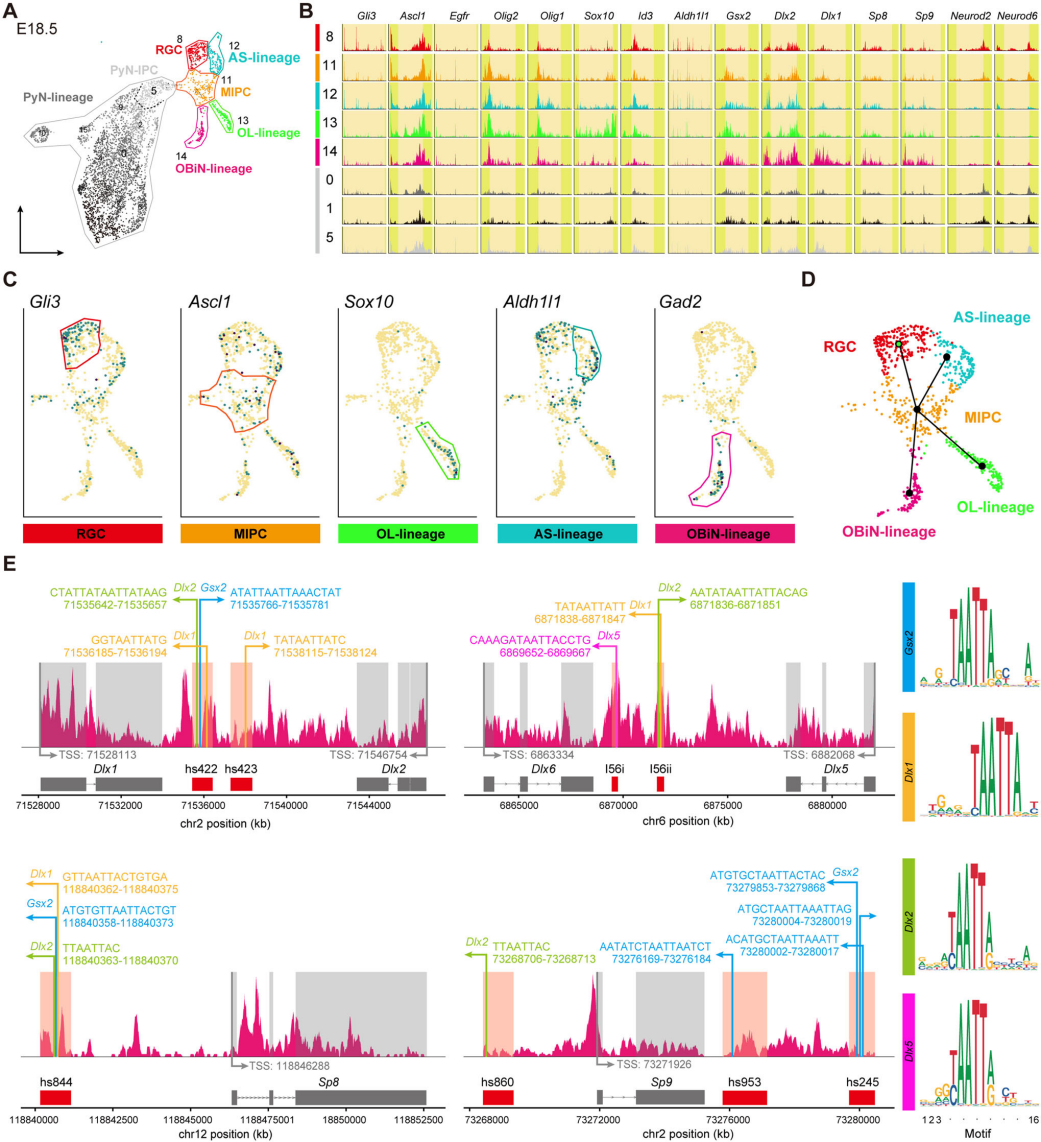
Delineation of cell types in E18.5 cortex and identification of master regulators of the OBiN-lineage. **A** Seventeen clusters (C0-C16) identified and annotated according to marker gene accessibility (scATAC-seq was performed on GFP^+^ cells from the E18.5 cortex of hGFAP-GFP transgenic mice). **B** Genome tracks showing normalized accessibility for marker genes with cluster-specific accessibility in their promoter and gene body. **C** UMAP plots showing distinct chromatin accessibility profiles of marker genes. **D** Scheme for construction of the developmental trajectory. **E** Enrichment analysis for transcription factor motifs in the OBiN-lineage showing that GSX2 and DLX1/2 bind to the VISTA enhancers of *Dlx1/2, Dlx5/6* and *Sp8/9*. The height of the letters (right panel) represents the frequency of each base in the cognate motif.

Dynamic changes in chromatin accessibility coincide with important aspects of glial and neuronal fate specification and differentiation. To characterize the chromatin state landscape of cortical cells in late embryogenesis, we used a transgenic mouse line that expresses GFP under the human GFAP promoter [16], in which GFP proteins carry over from RGCs to their immediate progeny. We performed scATAC-Seq on FACS-sorted GFP^+^ cells from the E18.5 hGFAP-GFP cortex. We generated an scATAC-Seq dataset from 4,721 cells passing quality control criteria. Integration of the promoter and gene body accessibility in scATAC-Seq together with marker gene expression in our scRNA-Seq and immunostaining results led to the identification of 17 clusters (C0-C16), including RGCs, MIPCs, AS-lineage, OL-lineage, OBiN-lineage, and PyN-lineage cells (Fig. 8A-C). For example, we found OBiN-lineage cells with accessibility at the gene loci of OBiN markers, including *Gsx2, Dlx1/2/5/6, Sp8/9,* and *Gad2/1* (Figs 8C, S4D). Indeed, all lineages analyzed in this study were consistent with the accessibility patterns at their marker gene loci (Fig. S4, S5), demonstrating the power of this technology.

Based on chromatin accessibility changes, we constructed developmental trajectories of all cell populations except the PyN-lineage. This analysis provided a clear trajectory of RGCs giving rise to MIPCs, which in turn generate AS-IPCs, OPCs, and OBiN-IPCs (Fig. 8D), consistent with our scRNA-Seq and intersectional genetic lineage-tracing analyses. Chromatin state profiling also provides a unique opportunity to characterize cell fate decisions underlying the emergence of cell types during development. The homeobox transcription factor genes *Dlx1* and *2* are central and essential components in the transcriptional code for OBiN development [20]. We identified multiple loci featuring DLX binding in known VISTA enhancers of *DLX1/2/5/6* and *Sp8/9* genes (Fig. 8E), consistent with previous DLX ChIP-Seq studies [32]. Most importantly, we found that GSX2 might also bind to these enhancers (Fig. 8E), providing evidence that GSX2 is at the top of the hierarchical gene regulatory network that governs OBiN development [23, 41–43]. Transcriptional and epigenetic regulation of oligodendrocyte development has been well studied [44, 45]. Our scATAC-Seq data identified chromatin accessibility of the oligodendrocyte developmental regulatory program in OL-lineage cells (Fig. S4E), and found that OLIG2 occupied *Sox10* conserved enhancers containing E-box motifs (CANNTG) (Fig. S4F) [46, 47]. Consistent with the accessibility patterns at astrocyte marker gene loci (Fig. S5A-C), we found that BMP signaling and NOTCH signaling might regulate astrocyte specification through promoting *Id* gene expression (Fig. S5D) and repressing *Ascl1* expression (Fig. S5E), respectively. ID proteins suppress ASCL1 functions through heterodimer formation, and HES1 actively and passively inhibits *Ascl1* expression [48]. Our analysis also revealed that HES1 may repress OL-lineage and OBiN-lineage gene expression to safeguard the specification and differentiation of astrocytes at each stage of development. Overall, our scATAC-Seq data provide evidence that modifications in chromatin accessibility contribute to the transcriptional control regulating the specification of cortical glia and OBiN.

## Discussion

**Fig. 9:**
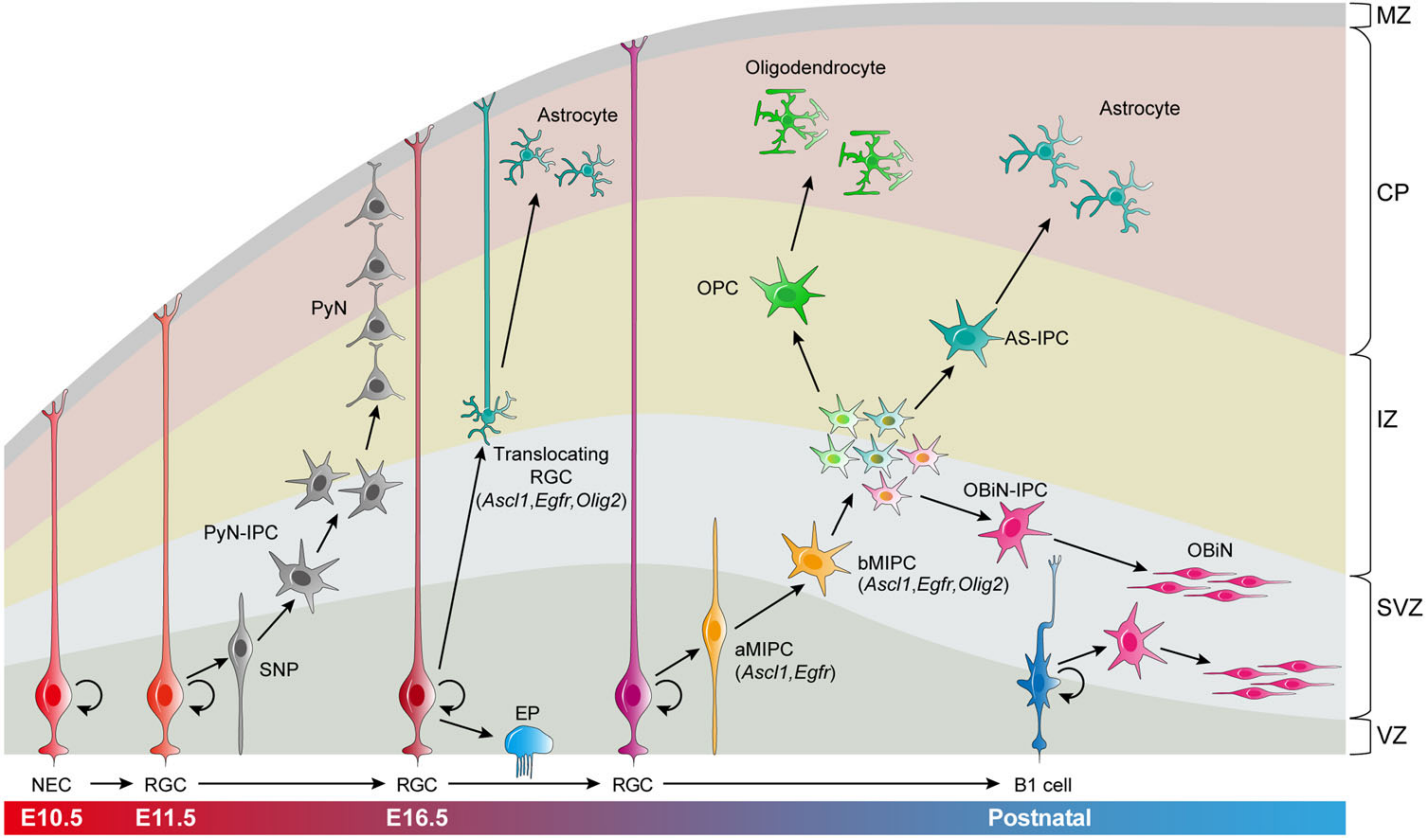
Summary of mouse cortical RGC lineage progression and the origins of cortical astrocytes, oligodendrocytes, and OBiNs. Neuroepithelial cells (NECs) convert into radial glial cells (RGCs) as the developing pallium (cortex) thickens. During E11.5-E16.5, cortical RGCs undergo asymmetric cell division to self-renew, and to produce short neural precursors (SNPs) and intermediate progenitor cells of pyramidal neurons (PyN-IPCs). PyN-IPCs exclusively generate PyNs. Around E16.5, cortical RGCs undergo lineage specification changes. Some RGCs begin to detach from the apical side and transform into astrocyte IPCs (AS-IPCs, translocating RGCs) that express EGFR, OLIG2, and ASCL1; some convert into ependymal cells (EPs). Meanwhile, RGCs begin to generate apical multipotent IPCs (aMIPCs) that express ASCL1 and EGFR. aMIPCs quickly differentiate into basal MIPCs (bMIPCs) that express ASCL1, EGFR, and OLIG2. bMIPCs undergo several rounds of divisions to generate oligodendrocyte progenitor cells (OPCs), AS-IPCs, and olfactory bulb interneuron IPCs (OBiN-IPCs). These lineage-restricted IPCs then divide symmetrically to generate cortical astrocytes, oligodendrocytes, and OBiNs. In neonatal and young adult mice, cortical RGCs/NSCs (B1 cells) mainly give rise to OBiN-IPCs, which in turn generate immature OBiNs that tangentially migrate into the OB. CP, cortical plate; IZ, intermediate zone; MZ, marginal zone; SVZ, subventricular zone; VZ, ventricular zone.

RGCs in the mouse prenatal neocortex progressively generate different subtypes of PyNs between E11.5-E16.5. Following that, RGCs have a fundamental change in their developmental potential and produce cortical oligodendrocytes, astrocytes, and OBiNs. Here, we show that, around E16.5, cortical RGCs first generate ASCL1^+^EGFR^+^ aMIPCs in the VZ and SVZ. The aMIPCs quickly differentiate into ASCL1^+^EGFR^+^OLIG2/1^+^MKI67^+^ bMIPCs, which undergo several rounds of divisions to generate OPCs, AS-IPCs, and OBiN-IPCs. These lineage-restricted IPCs then divide symmetrically to generate cortical astrocytes, oligodendrocytes, and OBiNs (Fig. 9). Interestingly, RGCs that are translocating to the cortex and transforming into AS-IPCs also express ASCL1, EGFR, and OLIG2. Finally, our scATAC-Seq data support our model for the gene regulatory logic and lineage progression underlying the specification and differentiation of cortical oligodendrocytes, astrocytes, and OBiNs (Fig. 9).

### Cortical RGC Lineage Progression

The results presented here provide novel evidence how cortical glial cells are generated from RGCs. Briefly, early cortical RGCs generate PyN-IPCs, which exclusively give rise to PyNs [6, 12, 49]. At the end of cortical PyN neurogenesis, around E16.5, RGCs switch lineages and produce aMIPCs/bMIPCs, which in turn generate cortical OPCs, AS-IPCs, and OBiN-IPCs (Fig. 9). This process is similar to that in the developing *Drosophila melanogaster* brain, where Type II neuroblasts divide to self-renew and to generate an immature intermediate progenitor. This progenitor undergoes a period of maturation, without cell division, to form a mature progenitor, after which mitosis recommences, and it generates neurons or glia [50].

SHH signaling plays a major role in modifying the output of cortical RGCs [51, 52]. During the period of cortical neurogenesis (E11.5-E16.5), VZ expression of GLI3R antagonizes SHH signaling. Loss of *Gli3* function results in ventralization of the cortex [13, 53–55]. As cortical PyN neurogenesis proceeds, SHH proteins secreted from migrating cortical interneurons and cells in the choroid plexus [36] promote changes the identity of cortical RGCs around E16.5. This transition is associated with reduced GLI3R levels, and an increase in *Ptch1*, *Smo*, and *Gli1* [13]. Cortical RGCs also start to express astrocyte marker genes including *Aldh1l1*, *Aqp4*, *GFAP, Slc1a2*, and *Tnc*. Beginning at this transition, cortical RGCs generate aMIPCs/bMIPCs, which give rise to both glia and OBiNs. The data support our model that, during this transition, cortical RGCs and IPCs take on properties similar to more ventral regions of the telencephalon. This is based on the expression of the *Ascl1*, *Dlx2/1, Egfr, Gsx2*, *and Olig2* genes and the process of GABAergic neurogenesis and gliogenesis [56, 57]. In addition to SHH, FGF signaling may also promote the RGC fate transition [51, 52].

Previous lineage tracing studies using *Ascl1-Cre* and *Ascl1-CreERT2* showed that *Ascl1*^*+*^ cells give rise to cortical oligodendrocytes and OBiNs, but not astrocytes [58]. This inconsistency appears to be due to unfaithfully-reproduced native gene expression by *Ascl1* transgenic lines, whereas *Ascl1-Flpo* and *Ascl1-CreER* knockin mice were used in this study. After birth, cortical RGCs/NSCs mainly give rise to OBiNs. This appears to be promoted by NOGGIN blocking endogenous BMP signaling [59]. After birth, cortical RGCs/NSCs generate a minority of cortical oligodendrocytes and astrocytes [60]; the major source of postnatally-generated glia is the local proliferation of OPCs [45] and AS-IPCs [19]. Notably, postnatal NSCs retain the ability to produce glial cells under specific circumstances. For example, when EGF is infused into the lateral ventricle, adult SVZ NSCs are induced to generate many astrocytes and a large number of myelin-forming oligodendrocytes [61, 62]. Neurosphere assays provide another good example [63].

### Cell Fate Specification of Cortical Oligodendrocytes

In the present study, based on specific molecular features and genomic sites of chromatin accessibility, we obtained evidence for the transcriptional pathways that control the generation of cortical OL-lineage, AS-lineage, and OBiN-lineage cells in the neonatal cortex. At the late embryonic stage, oscillation of *Hes1* and an increase in SHH signaling in cortical RGCs result in upregulation of *Ascl1* and *Egfr* in aMIPCs [13, 36, 55, 64, 65]. ASCL1 and EGFR may promote *Olig2/1* expression [66, 67]. Most important, an increase in SHH signaling strongly induces *Olig2/1* expression [68].

The hallmark of bMIPCs is co-expression of *Ascl1, Egfr*, and *Olig2/1*. Newly-born OPCs co-express *Ascl1, Egfr*, *Olig2/1, Sox10*, *and Pdgfra* (Fig. 2H), suggesting that cortical bMIPCs are biased toward OPC fates. While AS-IPCs maintain *Egfr* expression, OPCs quickly downregulate its expression. Thus, AS-IPCs are mainly dependent on EGFR-signaling, whereas OPCs are mainly dependent on PDGFRA-signaling for their expansion. *Ascl1, Olig2/1*, *Sox10*, and *Pdgfra* gene expression is essential for oligodendrogenesis [44, 45]. Recent studies described “Pre-OPCs” or “Pri-OPCs” in the developing mouse and human brain, an early progenitor cell type preceding committed OPCs [69–72]. The molecular features of the cortical “Pre-OPC” population resembles that of bMIPCs from this study, which are able to give rise to both cortical glial cells and OBiNs, suggesting that the “Pre-OPC” population might also be multipotent.

### Cell Fate Specification of Cortical Astrocytes

Cortical bMIPCs must downregulate *Ascl1* expression to differentiate into AS-lineage cells that continuously express *Egfr* and *Olig2/1* (Fig. 2I). What are the signals that drive the reduction of ASCL1 in cortical bMIPCs and promote the astrocyte fate specification? Our study suggests a mechanism related to the BMP and NOTCH signaling. BMP signaling is well known to induce the expression of the ID family in progenitor cells [73, 74]. ID proteins sequester the b-HLH dimerization partner and promote the degradation of ASCL1 protein [75, 76]. ID proteins also dimerize with HES1 to prevent its autorepression, increasing HES1 levels and inhibiting proneural gene expression [77, 78]. Moreover, OPCs and OBiN-IPCs, expressing ASCL1 and the Notch ligands *Dll1* and *Dll3* (Fig. S2A), activate NOTCH signaling in neighboring bMIPCs, conferring astrocytic differentiation potential on bMIPCs. NOTCH/HES1 activation is crucial for the fate specification, full differentiation, and quiescent state of astrocytes [48, 79]. Consistent with this notion, our scRNA-Seq and scATAC-Seq analyses showed that AS-lineage cells strongly expressed *Id1, Id2, Id3*, *Hes1,* and *Hes5* (Figs S2, S5). Cortical astrocytes have two origins: one from translocating RGCs and the other from bMIPCs; they both expressed ASCL1, EGFR, and OLIG2/1. Currently, we do not know the difference between these two groups of astrocytes.

The limited number of reliable markers to characterize AS-lineage cells at different developmental stages has impeded the study of astrocyte biology [80]. Here, we have identified stage-specific astrocyte markers. Our analysis revealed that *Egfr*^+^*Id3*^+^ cells are the earliest AS-IPCs, which in turn give rise to *Aldh1l1*^+^ AS-lineage cells, including AS-IPCs and immature and mature astrocytes. Notably, *Id3* is also expressed in a subset of cortical RGCs, PyN-IPCs, oligodendrocytes, pericytes, and endothelial cells (Figs 2D, 4E) [81, 82], as well as cortical interneurons [83]. Therefore, only EGFR^+^ID3^+^ cells can be recognized as AS-IPCs. Indeed, in our scRNA-Seq dataset from P1 brains, we have analyzed 10,003 cells and found that 1,418 expressed *Id3*. Among these *Id3*^+^ cells; 539 expressed *Egfr* and 186 expressed *Pdgfra*, but only 44 (3.1%) expressed both *Egfr* and *Pdgfra*, further supporting the notion that *Egfr*^*+*^*Id3*^*+*^ cells represent the AS-lineage. After birth, ALDH1L1 expression is a hallmark of astrocytes. Previously, it was reported that a subpopulation of ZBTB20^+^OLIG2^+^ cells that did not express SOX10 were astrocyte precursors in the postnatal cortex [84]. However, our scRNA-Seq analysis does not support this conclusion; nearly all RGCs and their progeny, including OL-lineage cells, expressed *Zbtb20* (Fig. S2C). We also found that *Nfia, Nfib*, and *Nfix* were widely expressed in P1 cortical cells (Fig. S2C).

### Cell Fate Specification of Cortical bMIPC-derived OBiNs

Although RGCs/NSCs in different domains along the lateral ventricle generate distinct types of OBiNs at late embryonic and postnatal stages [1], the development of most OBiNs is regulated by the gene regulatory network *Gsx1/2* – *Dlx1/2/5/6* – *Sp8/Sp9* – *Tshz1* – *Prokr2*; mutations in these genes result in loss of most OBiNs [20–24, 85, 86]. Increased SHH signaling induces *Gsx2* expression in some bMIPCs, while GLI3R may indirectly repress the *Gsx2* expression in the cortex through maintaining *Dmrta2*, *Dmrt3, Emx1*, *and Emx2* expression in the cortical VZ [53, 87]. GSX2 induces *Dlx1* and *2* expression [23, 42], which control the transcriptional programs crucial for telencephalic GABAergic neuronal development [32]. Specifically, DLX1 and 2 induce the expression of *Dlx5/6*, *Sp8/9*, *Gad1/2*, *Arx*, and *Etv1* [20, 41], key regulators of OBiN genesis. Moreover, GSX2 and DLX2 repress OPC specification [56, 88]. Thus, cortical bMIPCs that express *Gsx2* and *Dlx1/2* are biased toward OBiN differentiation. However, it is worth noting that *Gsx2* and *Dlx1/2* expression does not represent an irreversible state of OBiN commitment, as a small number of such cells also give rise to cortical astrocytes and/or oligodendrocytes [13, 89]. With the accumulation of DLX1/2 proteins and the induction of *Dlx5/6* and *Sp8/9*, bMIPCs differentiate into OBiN-IPCs, and their neuronal fate is determined. Consistent with this, *Dlx5/6-Cre* lines only label GABAergic neurons, but not oligodendrocytes or astrocytes [90, 91]. In the subpallium, DLX1 and 2 also promote GABAergic neuron production through repressing Olig2/1 [56, 92].

Taken together, these findings extend our comprehension of the mouse cortical RGC lineage and the developmental origins of cortical glia. Striking similar expression patterns of *Ascl1*, *Egfr, Dlx1/2, Gsx2*, and *Olig2* in the mouse cortex, septum, and striatum, and in the developing monkey and human cortex [70, 93, 94] suggests that mammals share a common molecular mechanism for making oligodendrocytes, astrocytes, and OBiNs. Through analysis of single-cell chromatin accessibility, we further identified the gene regulatory logic underlying the fate specification of cortical glial cells and OBiNs. This may also provide important insights into the cellular origin of malignant glioma, the most common primary brain tumor.

## Supplementary Information

Below is the link to the electronic supplementary material.

Supplementary material 1 (PDF 6120 kb)
