# Supplementary material for: Decoding Cortical Glial Cell Development
Source: Neurosci Bull. 2021 Feb 19;37(4):440–60. doi: 10.1007/s12264-021-00640-9 (PMC8055813; doi:10.1007/s12264-021-00640-9)
Supplement: Supplementary file 1 — Supplementary material 1 (PDF 6120 kb) [file 12264_2021_640_MOESM1_ESM.pdf]

Supplementary Materials

Title: Decoding Cortical Glial Cell Development

Xiaosu Li, Guoping Liu, Lin Yang, Zhenmeiyu Li, Zhuangzhi Zhang, Zhejun Xu, Yuqun Cai, Heng Du, Zihao Su, Ziwu Wang, Yangyang Duan, Haotian Chen, Zicong Shang, Yan You, Qi Zhang, Miao He, Bin Chen, Zhengang Yang

Supplemental Figures S1-S5

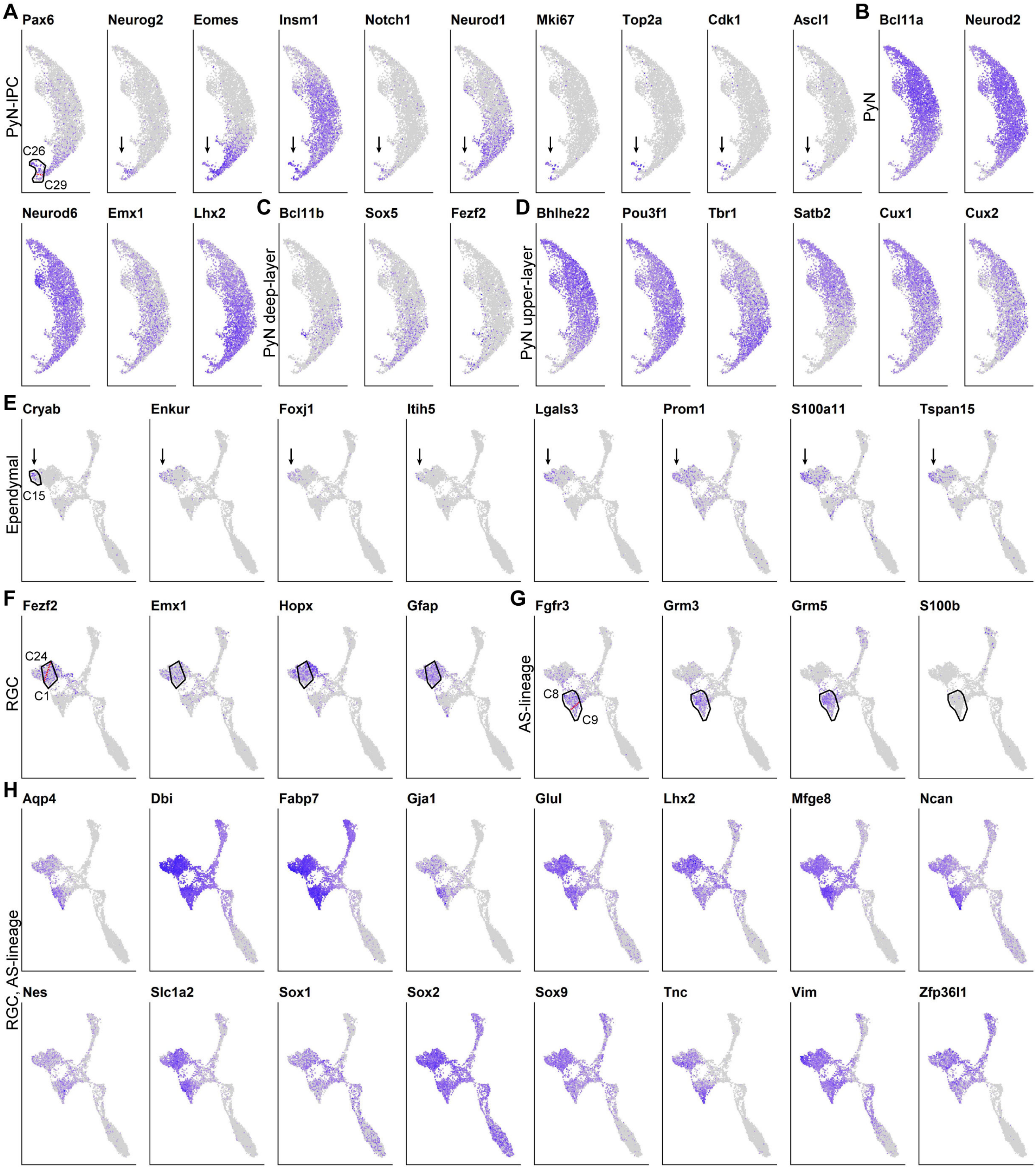

Fig. S1 Feature plots showing the cluster-specific expression of marker genes in PyN-lineage, ependymal cells, RGCs and AS-lineage. **A-D** Expression of marker genes in PyN-IPCs, deep and upper layer PyNs. **E** Expression of marker genes in immature ependymal cells. **F-H** Expression patterns of RGC and AS-lineage marker genes.

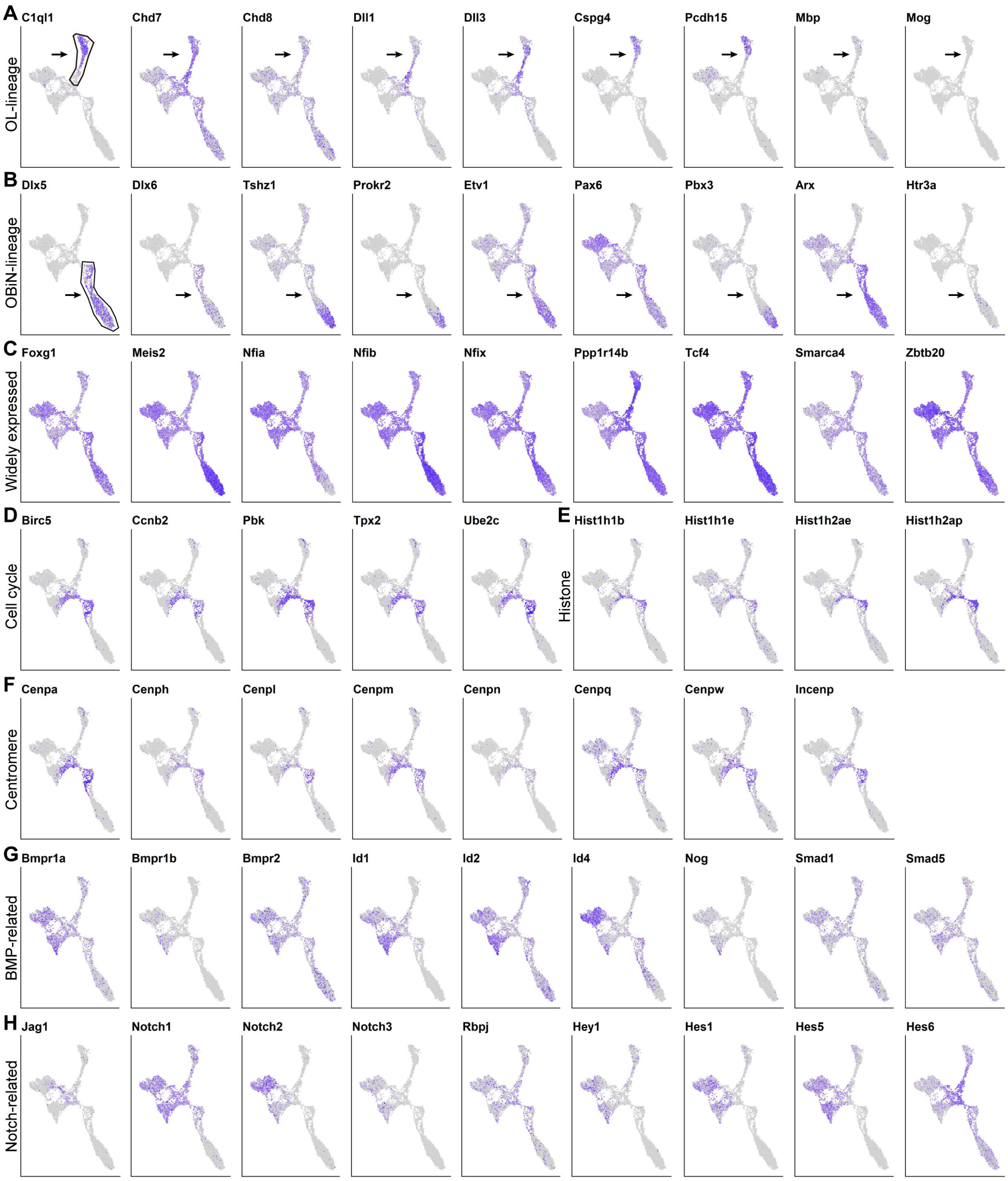

Fig. S2 Feature plots showing the expression patterns of genes. **A, B** Expression of marker genes in OL-lineage and OBiN-lineage. **C** Genes that widely expressed in RGC progeny. **D-F** Expression patterns of cell cycle, histone and centromere genes. **G, H** Expression patterns of BMP signaling- and Notch signaling- related genes.

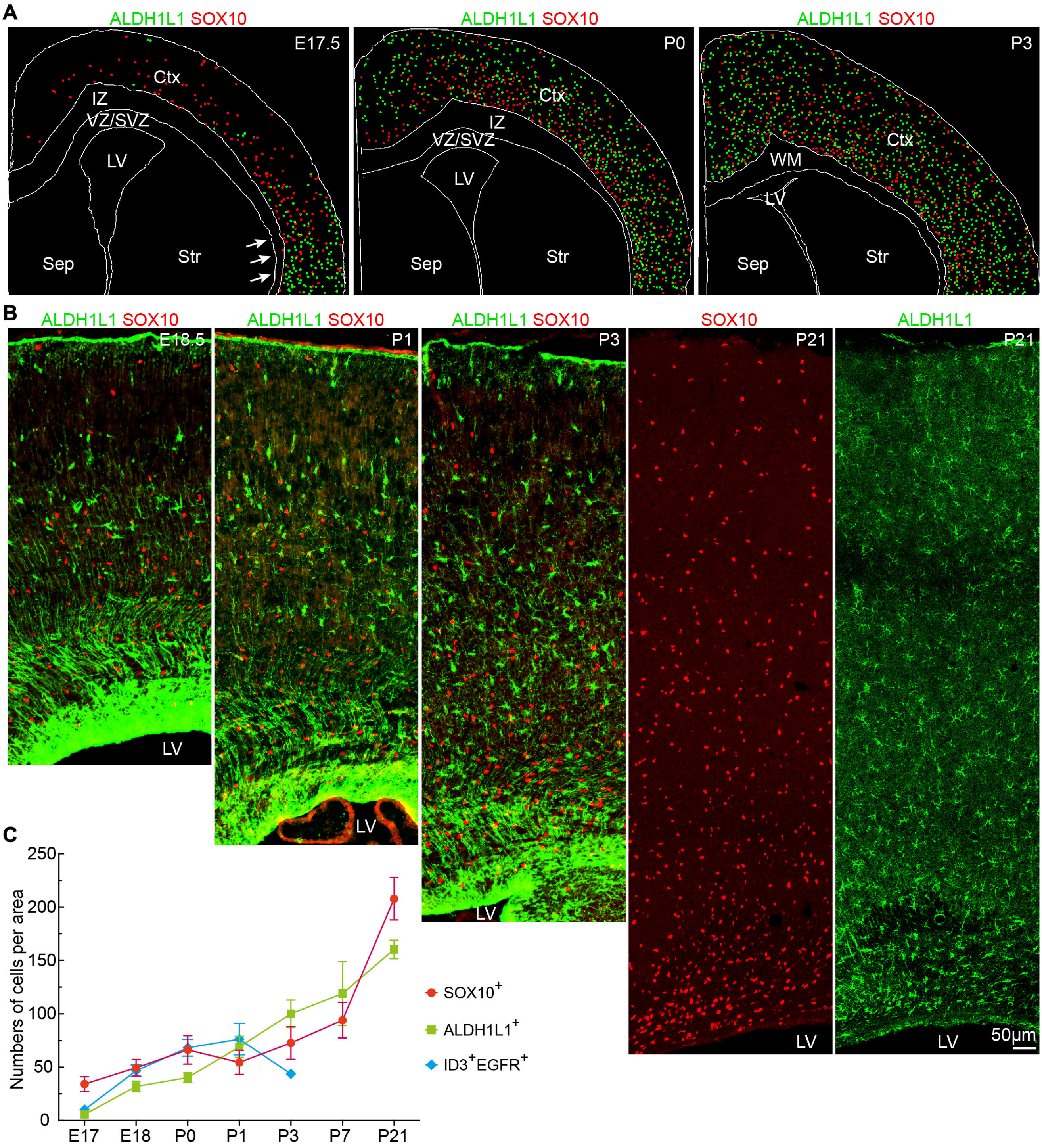

Fig. S3 The development of oligodendrocytes and astrocytes in the cortex. **A** Representative image showed ALDH1L1$^+$ and SOX10$^+$ cells in the cortex. Each dot represents one cortical glial cell. Note many more ALDH1L1$^+$ and SOX10$^+$ cells in the ventral cortex (arrows) at E17.5. **B** ALDH1L1$^+$ and SOX10$^+$ cells in the somatosensory cortex. **C** The numbers of SOX10$^+$, ALDH1L1$^+$, and EGFR$^+$ID3$^+$ cells in the somatosensory cortex. We quantified the number of cells in 12 μm thick, 350 μm width, brain coronal sections from similar rostral--caudal levels. We did not quantify EGFR$^+$ID3$^+$ cells in P7 and P21 cortex due to downregulation of EGFR expression. Means ± SEM.

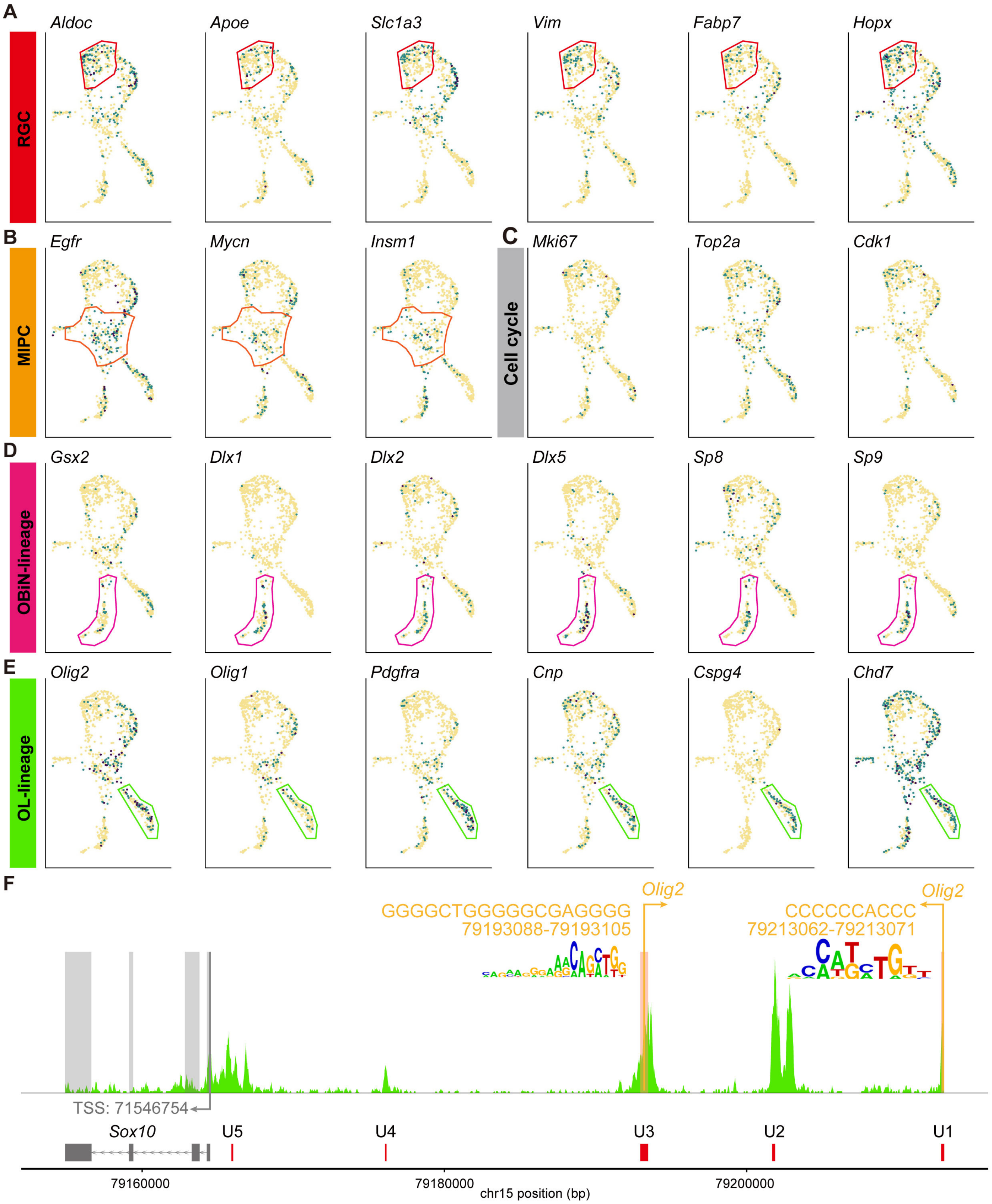

Fig. S4 UMAP plots showing distinct chromatin accessibility profiles of different cell types. **A-C** Chromatin accessibility of the RGC, MIPC and cell cycle marker genes. **D, E** Chromatin accessibility of the OBiN-lineage and OL-lineage regulatory programs. **F** OLIG2 bound to at least 2 conserved *Sox10* enhancers containing E-box (CANNTG) motifs.

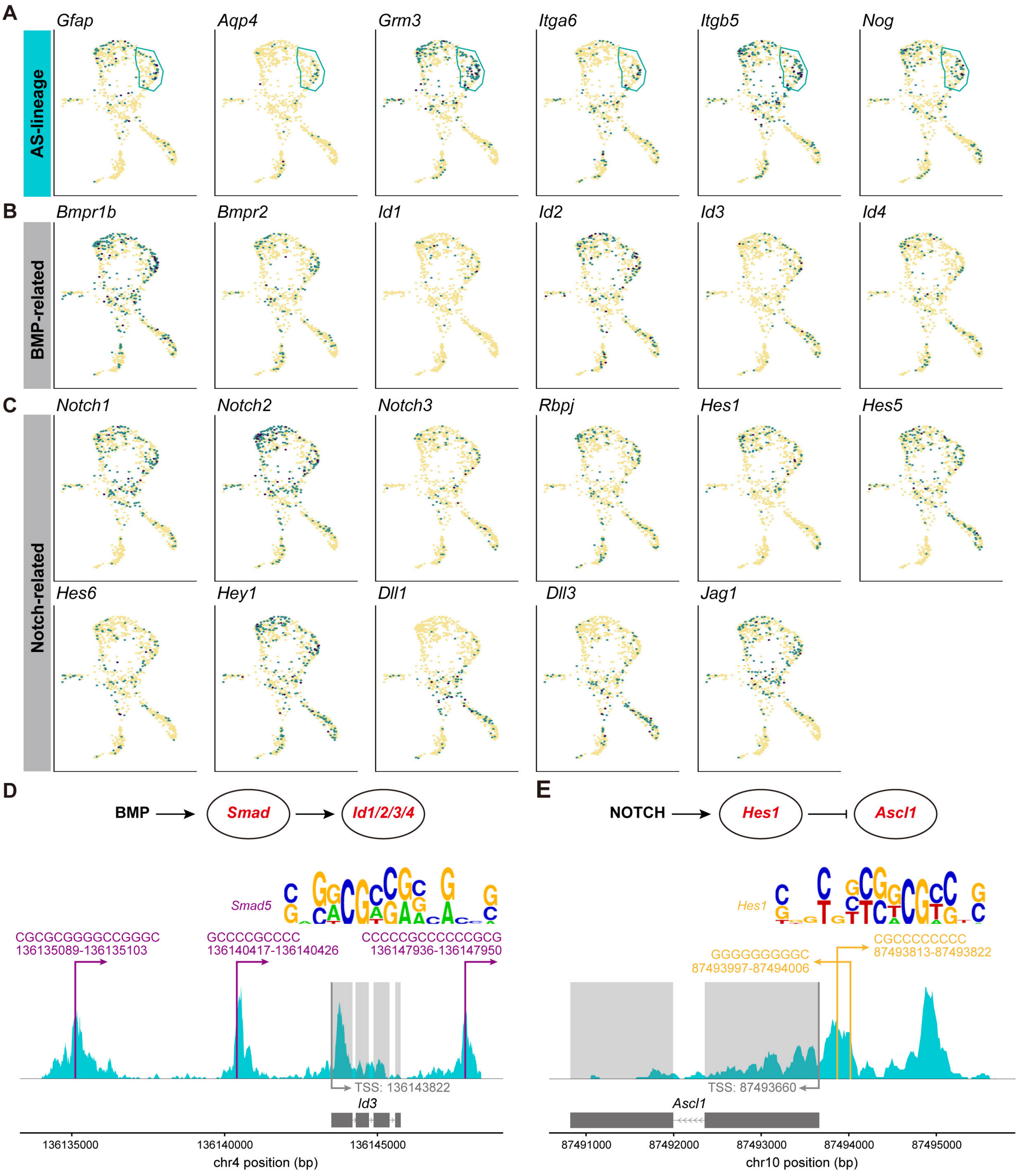

Fig. S5 UMAP plots showing distinct chromatin accessibility signatures of AS-lineage. **A-C** Chromatin accessibility profiles of AS-lineage genes, BMP- signaling and Notch-signaling related genes. **D** SMAD5 bound to enhancers of *Id3*. **E** HES1 bound to the promoter regions of *Ascl1*.
